# Neural substrates of brand equity: applying a quantitative meta-analytical method for neuroimage studies

**DOI:** 10.1016/j.heliyon.2022.e09702

**Published:** 2022-06-10

**Authors:** Shinya Watanuki, Hiroyuki Akama

**Affiliations:** a Department of Marketing, Faculty of Commerce, University of Marketing and Distribution Sciences, Kobe, Japan; b Institute of Liberal Arts/School of Life Science and Technology, Tokyo Institute of Technology, Tokyo, Japan

**Keywords:** Consumer neuroscience, Neuromarketing, DMN, Consumer decision making, ALE, Brand management, fMRI

## Abstract

Although the concept of brand equity has been investigated using various approaches, a comprehensive neural basis for brand equity remains unclear. The default mode network (DMN) as a mental process might influence brand equity related consumers' decision-making, as reported in the marketing literature. While studies on the overlapping regions between the DMN and value-based decision-making related brain regions have been reported in neuroscience literature, relationships between the DMN and a neural mechanism of brand equity have not been clarified. The aim of our study is to identify neural substrates of brand equity and examine brand equity-related mental processes by comparing them to the DMN. To determine the neural substrates of brand equity, we first carried out the activation likelihood estimation (ALE) meta-analysis. We examined 26 studies using branded objects as experimental stimuli for the ALE. Next, we set the output regions from ALE as the region of interest for meta-analytic connectivity modeling (MACM). Further, we compared the brand equity-related brain network (BE-RBN) revealed by the MACM with the DMN. We confirmed that the BE-RBN brain regions overlap with the medial temporal lobule (MTL) sub-system, a module composed of the DMN but excluding the retrosplenial cortex. Further, we discovered that several brain regions apart from the DMN are also distinctive BE-RBN brain regions (i.e., the insula, the inferior frontal gyrus, amygdala, ventral striatum, parietal region). We decoded the BE-RBN brain regions using the BrandMap module. The decoded results revealed that the brand equity-related mental processes are complex constructs integrated via multiple mental processes such as self-referential, reward, emotional, memory, and sensorimotor processing. Our study demonstrated that the DMN alone is insufficient to engage in brand equity-related mental processes. Therefore, marketers are required to make strategic plans to integrate the five consumer's multiple mental processes while building brand equity.

## 1 Introduction

Branded products and services have several advantages over unbranded ones. Consumers are willing to buy branded products at higher prices (Farquhar, 1989). They often buy branded products without deliberative thoughts (Hoyer and Brown, 1990) and have unconsciously favorable perceptions of them (Franzen et al., 2001). These behaviors tend to be habituated as long as they are satisfied with the products (Wood and Neal, 2009). Thus, most firms manage to add value to general products and services through branding, as this is an important source of financial profit for them (Farquhar, 1989).

What are the differences between branded and unbranded products? In marketing, these differences are attributed to “brand equity.” Brand equity can be understood as the strength of influence of a brand name as perceived by customers and the unique value premium that a company makes. Brands enjoying strong brand equity have a competitive edge when compared to their competitors and are therefore placed in an advantageous market position. Brand equity likely leads to higher consumer preferences, purchase intentions, and choice probabilities (Cobb-Walgren et al., 1995). Interbrand, which is a representative brand consulting firm, evaluated brand equity among dozens of brands and reported that Apple was considered the world's most valuable brand in 2020 (Interbrand, 2020). Apple produces a wide range of product categories and has dominant positions in these markets. Apple's success reveals that brand equity has been a crucial concept in marketing strategies for many decades.

The method of building brand equity has been theoretically and practically researched for three decades. Aaker (1992) organized the brand equity elements into five factors: brand associations, brand name awareness, brand loyalty, perceived brand quality, and other proprietary brand assets such as patents, trademarks, and channel relationships. Keller (2001) defined brand equity as the “differential effect of brand knowledge on consumer response to the marketing of the brand.” Kapferer (2008) referred to brand equity as “a set of mental associations and relationships built up over time among customers or distributors.” Thus, brand equity is an intangible asset residing in consumer's minds unlike tangibles, such as factories and office buildings.

Over the past two decades, neuroscience approaches have been employed in marketing. Many consumer-neuroscience studies have provided important findings about brain regions related to consumers’ decision making toward branded objects. However, these brain regions seem to be distributed in a wide variety of areas and are poorly convergent. McClure et al. (2004) reported that the dorsolateral prefrontal cortex (DLPFC) and hippocampus are the brain regions related to strong brand equity when comparing brands with strong and weak equity. In studies on brand personality, which is a critical concept in brand associations, Chen et al. (2015) reported that the MPFC is indeed associated with brand personality. These studies indicated that memory processing plays a crucial role in brand equity-based decision making. Yoon et al. (2006) demonstrated that it was not the medial prefrontal cortex (MPFC) but the inferior frontal gyrus that is related to brand personality judgment and argued that the information processed between a person and brand personality is distinct. They focused on the influences of self-referential processing on brands with brand equity. Meanwhile, according to Schaefer and Rotte (2007), the activation of the ventral striatum (VS) was observed when investigating the brain regions related to favorable brands. Esch et al. (2012) showed that the globus pallidus is related to the preference for a brand with strong equity. They emphasized the involvement of reward processing in brand equity-related mental processes. Reimann et al. (2011) showed the amygdala was associated with separation distress in regard to beloved brands. The amygdala is involved in emotional processing (LeDoux, 1992; Sergerie et al., 2008). This is the study focused on the emotional processing aspect of brand equity-related mental processes. Thus, although the mental processes corresponding to specified brain regions were examined in previous consumer neuroscience studies, few studies have assessed brand equity-related mental processes from the perspective of functional connectivity in the brain.

Moreover, brand equity-related mental processes, which may derive from the default mode network (DMN), are reported in the marketing literature. Numerous studies reported that the DMN is engaged in inward mental processes, such as recalling the past, wandering mental states, imagining the future, remembering autobiographical memory, self-related functions, scene construction, spontaneous thought, automated responses, decision-making based on system 1, and social cognition (Buckner et al., 2008; Northoff et al., 2006; Vatansever et al., 2017). For example, self-related functions are also one of the most important attributes of brand equity related mental processes. A self-expressive benefit is the most crucial value proposition derived from brand equity and is the most representative self-related concept on brand equity (Aaker, 1996). Spontaneous brand recall is an important metric for marketing strategies because it contributes significantly to financial performance (Kim et al., 2003). Spontaneous brand recall might be associated with spontaneous thought, a mental process derived from the DMN. Kervyn et al. (2012) noted the social cognitive aspects of brand equity. Consumers’ decision-making processes including the brand equity-based decision making are one of the behaviors derived from subjective value-based decision making. Several brain regions related to subjective value-based decision making, such as the ventral medial prefrontal cortex (VMPFC), MPFC, VS, medial temporal lobe (MTL), and posterior cingulate cortex (PCC), have been revealed by neuro imaging meta-analysis studies (Acikalin et al., 2017; Bartra et al., 2013; Sescousse et al., 2013). Clithero and Rangel (2014) noted resemblances between the DMN and value-based decision making with regard to these activated brain regions. Acikalin et al. (2017) demonstrated that most of the brain regions related to subjective value-based decision making overlapped with most of the brain regions comprising the DMN. Despite these resemblances between mental processes derived from two distinct sources, such as the DMN and brand equity, no studies have been conducted to assess the overlap of the DMN and brand equity-related regions of the brain.

Therefore, the aim of the present study is to reveal the distinct brain regions related to brand equity and to provide characteristics for brand equity-related mental processes in consumers’ decision making by comparing brain regions involved in both the brain equity-related network (BE-RBN) and the DMN through performing a quantitative meta-analysis of neuroimaging studies.

## 2 Materials & methods

First, to compare the brand equity-related brain regions with the DMN-related brain regions, we collected related foci data. Each activation likelihood estimation (ALE) map was created using an ALE method based on each focus. Second, meta-analytic connectivity modeling (MACM) was conducted by setting each cluster of the brand equity-related brain regions, which were produced by an ALE method, as a region of interest (ROI). Smith et al. (2009) pointed out that brain regions identified by MACM are almost equal to brain regions consisting of resting state networks. This means that brain regions identified by the MACM might represent brain regions comprising the BE-RBN. Last, we conducted a conjunction and contrast analysis between the BE-RBN and DMN to reveal overlap and each distinct brain region in both brain networks. We attempted to identify each characteristic mental process derived from these distinct brain regions in both brain networks. Moreover, we assessed publication biases on the ALE results about the brand equity-related brain regions using Fail-Safe N (FSN) analysis.

### 2.1 Data collection and an ALE method

**Figure 1:**
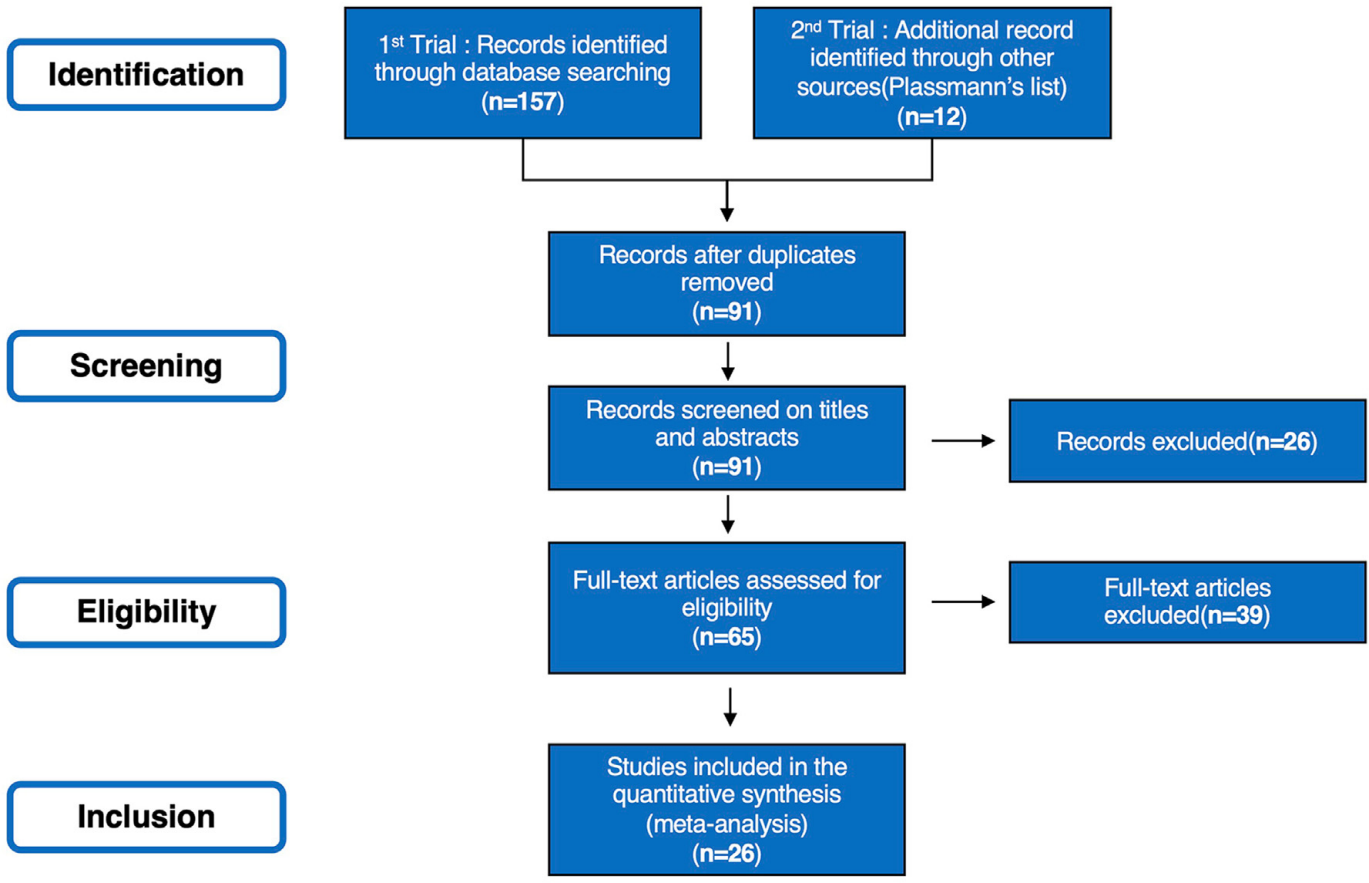
Prisma flow diagram.

First, a systematic literature search was performed to select neuro imaging studies on consumers' decision making on branded products and services. The search was conducted using the PubMed database (https://pubmed.ncbi.nlm.nih.gov). We included published studies between January 2000 and March 2021 from only peer-reviewed English language journals. We searched for studies using functional magnetic resonance imaging (fMRI) with the specific terms “brand,” “consumer,” “fMRI,” “neural,” “choice,” “purchase,” “decision-making,” and “preference.” This search processes yielded 10 for “brand, fMRI, neural, and choice”; 0 for “brand, fMRI, neural, and purchase”; 11 for “brand, fMRI, neural, and decision-making”; 12 for “brand, fMRI, neural, and preference”; 38 for “consumer, fMRI, neural, and choice”; 12 for “consumer, fMRI, neural, and purchase”; 48 for “consumer, fMRI, neural, and decision-making”; and 26 for “consumer, fMRI, neural, and preference.” We then added the studies listed in Plassmann's (Plassmann et al., 2012) literature lists on branding. Studies were selected according to the following inclusion criteria: (1) studies that conducted fMRI scans of healthy participants, (2) studies that were conducted in a consumption context, (3) studies where branded objects were used as experimental stimuli, for example, products, logo, advertising with brand logos, and (4) those that reported activations as three-dimensional coordinates in the stereotactic space of Talairach or the Montreal Neurological Institute (MNI). Moreover, in this study, we included studies for this meta-analysis, regardless of types of stimulus materials for experiments, such as foods, consumer package goods, and durable goods. We excluded studies that did not meet these criteria. However, there were a few exceptions to the inclusion and exclusion. Two studies (Klucharev et al., 2008; Plassmann et al., 2008) did not directly use branded objects as experimental stimuli. However, they were ultimately included in quantitative synthesis because these stimuli were regarded as objects similar to branded ones. These two studies were also included in Plassmann's lists (Plassmann et al., 2012). Additionally, we excluded Knutson et al. (2001), despite using products with logos as experimental stimuli. We considered the attractiveness of their stimuli equal to that of unbranded objects because they controlled for attractiveness between branded and unbranded products. The preferred reporting items for systematic reviews and meta-analyses (PRISMA) flow diagram (Figure 1) provide details of the screening process. Twenty-six studies were included in the present meta-analysis (Supplementary Table S1).

Second, for the DMN, foci were collected by searching relevant studies via Sleuth version 3.0.4 (https://www.brainmap.org/sleuth/). The search criteria were set according to Acikalin et al. (2017) as follows: (1) Context in Experiments is “Normal Mapping”; (2) Activation in Experiments is “Deactivation”; and (3) Control in Experiments is “Low level”. The matching results yielded 93 papers that included 185 experiments, 1,631 foci, and 1,510 subjects.

The entire activation foci in the Talairach space were converted to the MNI space via a transformation algorithm (Lancaster et al., 2007). Thus, these foci, selected across a wide variety of experiment conditions such as stimuli and task, were used in this study.

The ALE method (Eickhoff et al., 2009) was adopted for the following reasons. First, it is the most popular quantitative meta-analysis method (Acar et al., 2018). Second, the BrainMap platform (http://www.brainmap.org/) contains all the necessary tools for an ALE analysis (Fox et al., 2014; Fox and Lancaster, 2002). The GingerALE software calculates an ALE algorism based on the reported foci. Sleuth software is a tool for curating studies to match set conditions, such as experimental tasks, locations, and subjects. Mango software (http://ric.uthscsa.edu/mango/), an associated software of the BrainMap platform, is a visualization tool for the resulting ALE maps and various NIfTI files and implements a decoding analysis tool based on the BrainMap database. In addition to these tools, the BrainMap platform is equipped with a plethora of other functions for neuro imaging meta-analysis. Finally, given that significant brain imaging meta-analysis studies regarding subjective value-based decision-making have been conducted using ALE (Acikalin et al., 2017; Bartra et al., 2013; Clithero and Rangel, 2014; Sescousse et al., 2013), adopting an ALE method to assess our results may prove beneficial when comparing findings from these previous studies. It is a coordinate-based quantitative meta-analysis method, and its procedures are as follows. First, the modeled activation map was created by applying three-dimensional Gaussian probability density function to each individual focus. Similar procedures were conducted on all foci of selected studies. With increased convergence of reported foci, across studies, there has been a gradual minimization in the variance of Gaussian probability distribution. This means that the contingency of reported foci in each individual study is expected to be eliminated. Second, an ALE map was obtained by calculating the union of these modeled activation maps. Finally, to create a more accurate ALE map, it was compared with the randomness map created by null distribution. Concretely, the thresholded ALE map was obtained by conducting a permutation test for assessing differentiations between these maps at each voxel (Eickhoff et al., 2009; Turkeltaub et al., 2012). The ALE method was conducted using the GingerALE version 3.02 tool (http://www.brainmap.org/). The thresholding analyses were performed using a cluster-level correction for multiple comparisons at p = 0.05, with a cluster-forming threshold of p = 0.001. The permutation size was set to 1000. In the present study, coordinates were reported in the MNI space. The complete images with brain activations were output as NIfTI files and overlaid onto a canonical anatomical T1 brain template in MNI space using the Mango software (version 4.1; http://ric.uthscsa.edu/mango/).

### 2.2 MACM

MACM is a method to identify co-activation brain regions related to a set ROI by curating the studies in the BrainMap database (Laird et al., 2013; Robinson et al., 2010). The BrainMap has two kinds of databases: a functional database and a voxel-based morphometry (VBM) database. This study used a functional database, which contains 3,406 papers, 111 paradigm classes, 76,016 subjects, and 131,598 experiments. To calculate statistically significant co-activated brain regions with the set ROI, mathematical methods are executed based on curated foci. We adopted an ALE method (Turkeltaub et al., 2002), and used the GingerALE version 3.02 tool (http://www.brainmap.org/) to perform the calculation. The thresholding analyses were performed using a cluster-level correction for multiple comparisons at p = 0.05, with a cluster-forming threshold of p = 0.001. The permutation size was set to 1,000.

### 2.3 Conjunction and contrast analysis

The conjunction and contrast analysis between the BE-RBN and the DMN were conducted using the GingerALE version 3.02 tool (http://www.brainmap.org/). Different brain regions from these two networks were compared to brain regions generated from a null distribution according to statistically appropriate criteria. Thus, this analysis identified statistically significant overlap and each distinct brain region between the BE-RBN and the DMN. For this analysis, the thresholding criteria were set as follows: P-value = p < 0.05, number of permutations = 1,000, and minimum cluster size = 100 mm^3^.

### 2.4 Decoding analysis

We decoded both shared and each distinct brain region, BE-RBN and DMN, using the behavioral analysis plugin, a brain image analysis application provided by the BrainMap platform (Lancaster et al., 2012). This plugin tool is implemented in Mango software (version 4.1; http://ric.uthscsa.edu/mango/). This plugin tool enables the analysis of behaviors and cognitive functions regarding the set ROI. This tool was developed based on the BrainMap database. Foci corresponding with five behavioral domains (“Action”, “Cognition”, “Emotion”, “Interoception”, and “Perception”) are organized in the BrainMap database. Moreover, these behavioral domains are classified into sixty sub-domains. For example, the Action domain has eight sub-domains such as “Execution (Speech)”, “Execution (Unspecified)”, “Imagination”, “Inhibition”, “Motor Learning”, “Observation”, “Preparation”, and “Rest”. Thus, the sub-domain items are detailed profiles of behavioral domains. The detailed items of the sub-domains are listed on the BrainMap home page (https://brainmap.org/taxonomy/behaviors/). Given that each activated focus has a unique sub-domain tag, characteristic items of sub-domains corresponding with the set specific ROI can be calculated by significance testing. This is a method of comparing observed probabilities of sub-domain items within the set specific ROI with probabilities generated from the null distribution. Therefore, statistically significant behavioral and cognitive profiles within the ROI can be calculated. An item is considered significant if its z-score is above 3.0. This criterion was determined based on a bonferroni correction for multiple comparisons (p ≤ 0.05). Thus, because the correspondences between sub-domains and brain regions were statistically validated, the usage of the plugin can be expected to avoid a reverse inference problem (Lancaster et al., 2012). Given that the plugin is provided using the Talairach template, the ROI created on the MNI template is needed to transform the MNI space to Talairach space.

### 2.5 Fail-Safe N for the results of ALE on brand equity related brain regions

Although the coordinate-based meta-analysis (CBMA) such as ALE have rigorous methods, publication biases are important concerns. To assess this problem, we validated the publication biases of our ALE results for brand equity related brain regions by conducting FSN analysis. FSN refers to the maximum number of studies that alter results obtained by a meta-analysis. Thus, the larger the number of FSN, the more robust the results obtained by meta-analysis. According to Acar et al. (2018), there is an estimation for normal mapping in that a confidence interval with 95% for the number of studies that report no local maxima varies from 5 to 30 per 100 published studies. Thus, given that 94 experiments are contained in our study, unpublished experiments were estimated at 28 and the minimum FSN was also set as 28. We calculated the FSN in each cluster in our ALE results based on the procedure described by Acar et al. (2018).

## 3 Results

### 3.1 ALE

**Figure 2:**
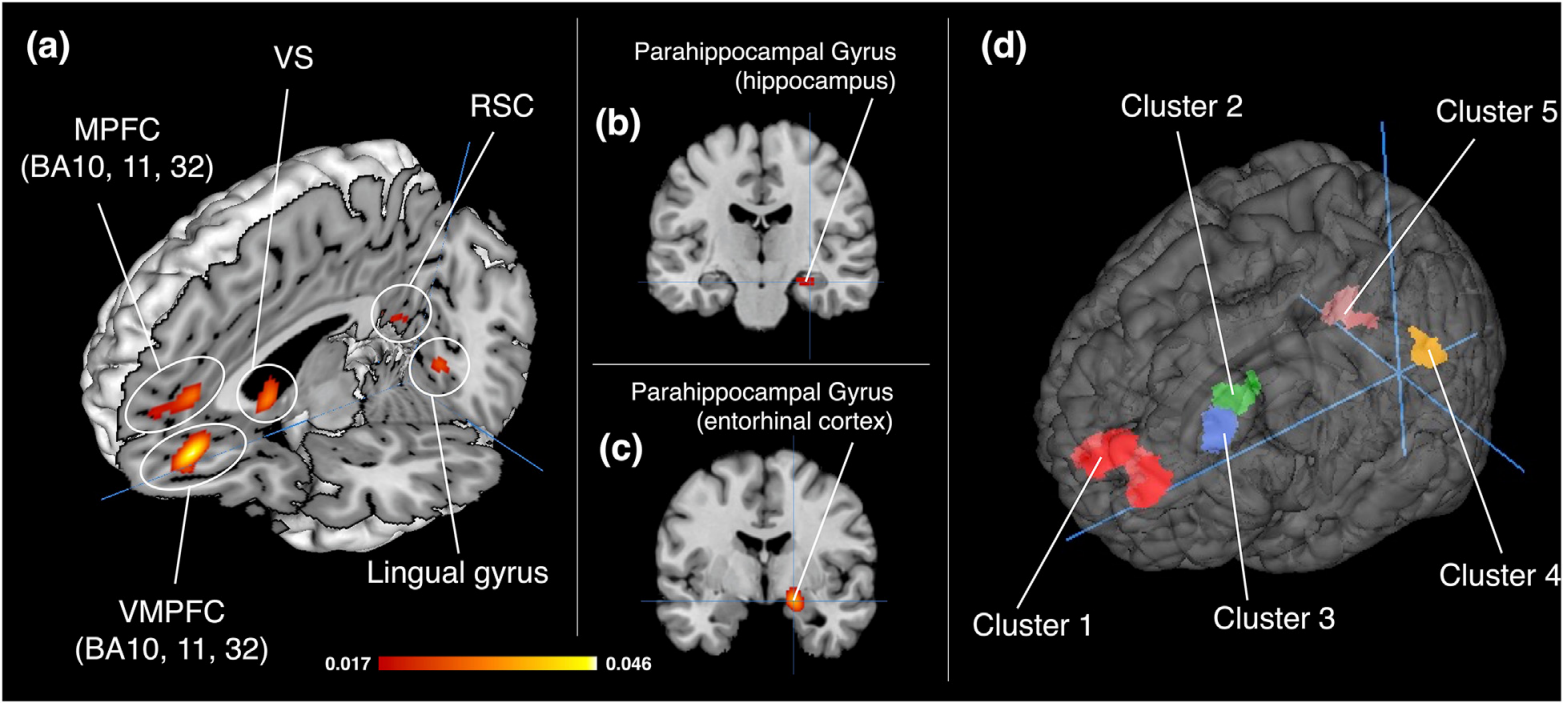
Results of activation likelihood estimation values for brand equity (cluster-level P < 0.05, cluster-forming threshold at voxel level: P < 0.001). (a) 3D view, Crosshair (-4, -70, -21), (b) Coronal view, Crosshair (30, -18, -18), (c) Coronal view, Crosshair (18, -4, -16)/**Abbreviations**; MPFC, medial prefrontal cortex; VMPFC, ventral medial prefrontal cortex; VS, ventral striatum; RSC, retrosplenial cortex. (d) ROIs for the meta-analytic connectivity modeling; Red = Cluster 1, Green = Cluster 2, Blue = Cluster 3, Brown = Cluster 4, Yellow = Cluster 5.

In terms of activation foci related to brand equity, the meta-analysis identified significant convergence in five clusters (Table 1; Figure 2-(a)(b) (c)). These clusters were located in the rostral anterior cingulate cortex (rACC, BA32, ventral MPFC [VMPFC]), the medial frontal gyrus (MFG, BA10), the parahippocampal gyrus (PHG, the entorhinal cortex <BA28>, hippocampus), the caudate head (the anterior part of ventral striatum), the posterior cingulate cortex <PCC> (the retrosplenial cortex <RSC>; BA29, BA30), and the lingual gyrus. These five clusters were used as ROIs for the MACM (Figure 2(d)).

**Table 1:** Regional foci of brain activation by the ALE (Brand equity related brain regions).

Cluster #	Side	Brain region	BA	Peak voxel coordinates (MNI)			ALE values	Cluster Size (mm^3^)
				x	y	z		
1	L	Anterior Cingulate (VMPFC)	32	-4	42	-16	0.046	6368
	L	Anterior Cingulate (MPFC)	32	-4	44	8	0.030	
	R	Anterior Cingulate (MPFC)	32	10	50	-6	0.027	
	L	Medial Frontal Gyrus (MPFC)	10	-10	52	10	0.023	
	L	Medial Frontal Gyrus (MPFC)	10	0	58	6	0.021	
2	R	PHG (Entorhinal cortex)	28	18	-4	-16	0.036	2216
	R	PHG (Hippocampus)	---	30	-18	-18	0.020	
3	L	Caudate Head (VS)	---	-6	12	-4	0.034	1936
4	R	Posterior Cingulate (Retrosplenial region)	30	6	-52	16	0.026	1064
	L	Posterior Cingulate (Retrosplenial region)	30	-6	-58	12	0.020	
	L	Posterior Cingulate (Retrosplenial region)	29	-4	-50	14	0.019	
5	L	Lingual Gyrus	18	-18	-74	-4	0.033	1032
	L	Lingual Gyrus	18	-6	-78	-2	0.019	

BA, Brodmann Area; MNI, Montreal Neurological Institute; ALE, activation likelihood estimation; L, Left; R, Right; MPFC, medial prefrontal cortex; VMPFC, ventromedial prefrontal cortex; VS, ventral striatum.

**Figure 3:**
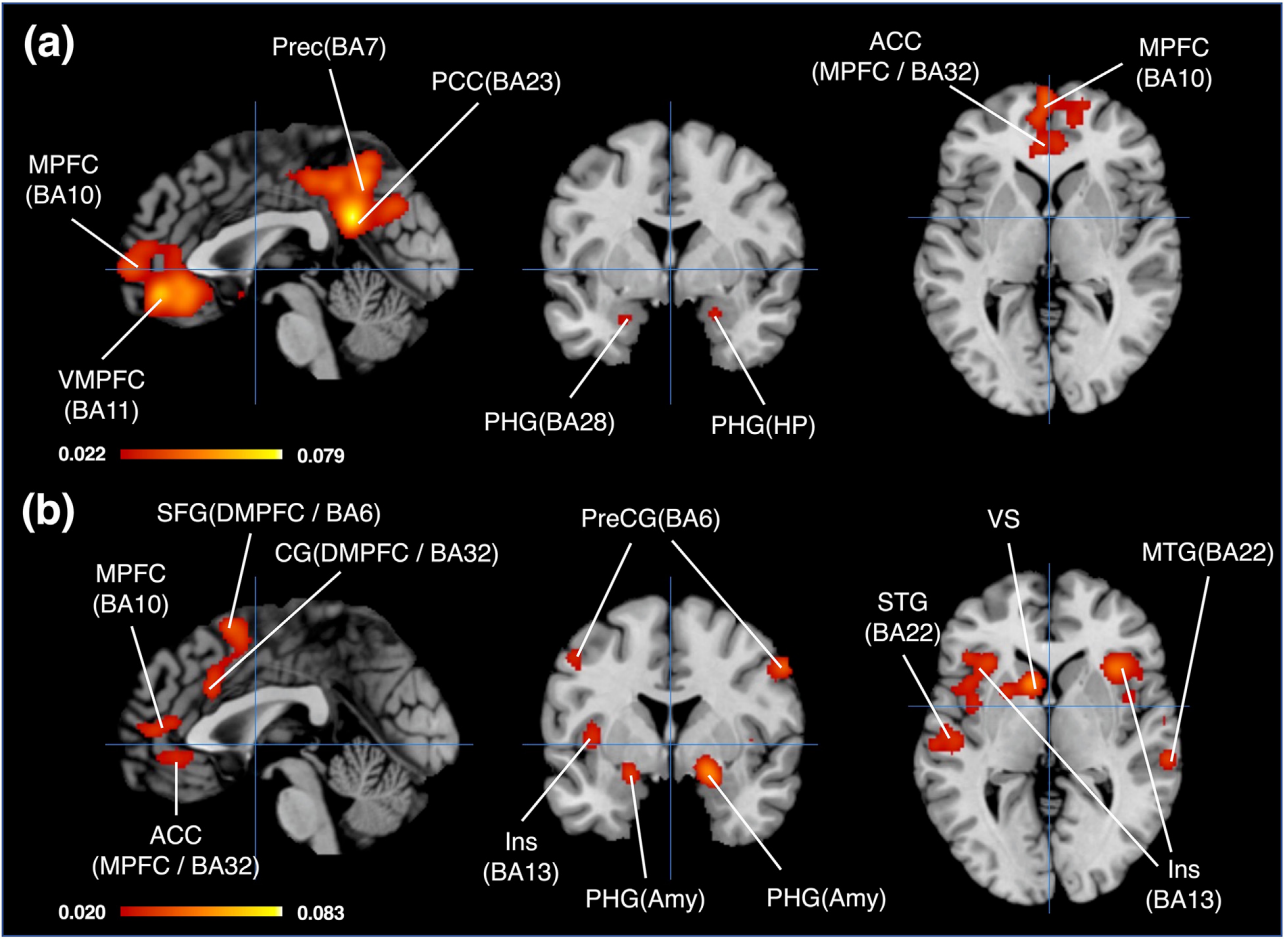
Activated brain regions of both the default mode network and the brand equity related brain network. (a) Brain regions of the default mode network, Crosshairs (0, 0, 0), (b) Brain regions of Brand equity related brain network, Crosshairs (0, 0, 0)/**Abbreviations**; ACC, anterior cingulate cortex; Amy, amygdala; BA, Brodmann area; CG, cingulate cortex; DMPFC, dorsal medial prefrontal cortex; HP, hippocampus; Ins, insula; MPFC, medial prefrontal cortex; MTG, middle temporal gyrus; PCC, posterior cingulate cortex; PHG, parahippocampal gyrus; PreC, precuneus; PreCG, precentral gyrus; SFG, superior frontal gyrus; STG, superior temporal gyrus; VMPFC, ventral medial prefrontal cortex; VS, ventral striatum.

For the DMN, significant activated brain regions were divided into nine clusters (Table 2; Figure 3(a)). Brain regions with the highest ALE value within each cluster were as follows: posterior cingulate cortex (PCC, BA23, Cluster1), rACC (BA32, VMPFC, Cluster2), inferior parietal lobule (IPL, BA40, Cluster3), middle temporal gyrus (MTG, BA39, Cluster4), PHG (amygdala, Cluster5), middle frontal gyrus (MFG, BA8, Cluster6), middle temporal gyrus (MTG, BA39, Cluster7), PHG (hippocampus, Cluster8), and middle frontal gyrus (MFG, BA8, Cluster9).

**Table 2:** Regional foci of brain activation by the ALE (DMN related brain regions).

Cluster #	Side	Brain region	BA	Peak voxel coordinates (MNI)			ALE values	Cluster Size (mm^3^)
				x	y	z		
1	L	Posterior Cingulate	23	0	-50	26	0.0792	26472
	L	Precuneus	7	0	-56	46	0.0541	
	L	Cingulate Gyrus	31	2	-42	44	0.0469	
	R	Cingulate Gyrus	31	4	-36	46	0.0444	
	L	Precuneus	7	-2	-58	56	0.0439	
	R	Precuneus	31	4	-72	34	0.0410	
	L	Precuneus	31	-4	-68	28	0.0403	
	R	Precuneus	31	14	-58	26	0.0355	
	R	Cingulate Gyrus	31	10	-26	40	0.0311	
	R	Paracentral Lobule	31	4	-18	48	0.0286	
2	R	Anterior Cingulate (VMPFC)	24	4	34	-14	0.0701	22224
	L	Anterior Cingulate (VMPFC)	32	0	48	-12	0.0648	
	L	Medial Frontal Gyrus (MPFC)	10	-2	64	0	0.0431	
	R	Caudate Head (VS)	---	8	12	-12	0.0361	
	L	Medial Frontal Gyrus (MPFC)	9	-2	58	8	0.0352	
	R	Medial Frontal Gyrus (MPFC)	10	14	52	-2	0.0300	
	R	Anterior Cingulate (MPFC)	32	4	40	8	0.0298	
	R	Medial Frontal Gyrus (MPFC)	10	20	60	2	0.0292	
	L	Anterior Cingulate	25	-4	6	-10	0.0255	
3	R	Inferior Parietal Lobule	40	60	-30	38	0.0464	3456
	R	Inferior Parietal Lobule	40	58	-26	24	0.0447	
4	R	Middle Temporal Gyrus	39	50	-68	20	0.0492	3016
	R	Angular Gyrus	39	50	-72	36	0.0289	
	R	Superior Temporal Gyrus	22	60	-56	20	0.0237	
	R	Middle Temporal Gyrus	39	50	-60	28	0.0232	
5	L	PHG (Amygdala)	---	-26	-6	-24	0.0395	2888
	L	PHG (Entorhinal cortex)	28	-22	-16	-16	0.0390	
6	L	Middle Frontal Gyrus	8	-26	24	44	0.0478	2536
7	L	Middle Temporal Gyrus	39	-42	-72	22	0.0321	2520
	L	Middle Temporal Gyrus	19	-40	-82	32	0.0271	
8	R	PHG (Amygdala)	---	30	-14	-18	0.0355	1616
	R	PHG (Entorhinal cortex)	---	24	-2	-22	0.0301	
9	R	Middle Frontal Gyrus	8	32	28	42	0.0332	1224
	R	Middle Frontal Gyrus	8	28	28	40	0.0332	

BA, Brodmann Area; MNI, Montreal Neurological Institute; L, Left; R, Right; MPFC, medial prefrontal cortex; VMPFC, ventromedial prefrontal cortex.

### 3.2 MACM

To identify BE-RBN, we conducted MACM based on brand equity-related brain regions revealed by the ALE. We created the five ROIs according to the five clusters of brand equity-related brain regions using Mango software. Foci relevant to each ROI were curated via the BrainMap database using the Sleuth software. The search criteria were as follows: (1) Context in Experiments is “Normal Mapping”; (2) Activation in Experiments is “Deactivation”; (3) Control in Experiments is “Low level”; and (4) MNI image in Location is “each ROI corresponding to each cluster” that we created. The matching results in cluster1 yielded 19 papers that included 23 experiments, 448 foci, and 365 subjects. The matching results in cluster2 yielded 18 papers that included 22 experiments, 353 foci, and 323 subjects. The matching results in cluster3 yielded 14 papers that included 14 experiments, 325 foci, and 254 subjects. The matching results in cluster4 yielded 2 papers that included 2 experiments, 25 foci, and 59 subjects. The matching results in cluster5 yielded 8 papers that included 8 experiments, 195 foci, and 116 subjects. The MACM input data were constructed by aggregating these foci. After two duplicated foci were eliminated, 1,297 foci were adopted as the MACM input data. The MACM results are shown in Table 3 and Figure 3(b).

**Table 3:** Regional foci of brain activation by the MACM (Brand equity-related brain network).

Cluster #	Side	Brain region	BA	Peak voxel coordinates (MNI)			ALE values	Cluster Size (mm^3^)
				x	y	z		
1	L	Caudate Body	---	-10	12	2	0.0555	10208
	L	Insula	---	-32	24	-4	0.0432	
	L	Insula	13	-44	12	-4	0.0361	
	L	Inferior Frontal Gyrus	47	-44	26	-2	0.0338	
	L	Insula	13	-34	16	6	0.0329	
	L	Putamen	---	-22	8	0	0.0326	
	L	Insula	13	-40	2	4	0.0315	
	L	Insula	13	-50	10	4	0.0301	
2	R	Claustrum	---	36	20	0	0.0481	5760
	R	Precentral Gyrus	44	46	10	4	0.0283	
	R	Insula	13	52	10	-8	0.0275	
3	L	Superior Frontal Gyrus (DMPFC)	6	2	12	60	0.0421	5456
	L	Medial Frontal Gyrus (DMPFC)	6	-4	6	52	0.0412	
	L	Cingulate Gyrus (DMPFC)	32	0	22	30	0.0339	
4	R	PHG (Amygdala)	---	20	-4	-14	0.0827	3304
5	L	Medial Frontal Gyrus	9	-2	50	8	0.0339	2928
	R	Anterior Cingulate (MPFC)	24	6	38	12	0.0321	
	L	Anterior Cingulate (MPFC)	32	-2	44	10	0.0311	
6	L	Precentral Gyrus	4	-50	-6	44	0.0458	2808
	L	Precentral Gyrus	6	-42	-4	58	0.0244	
	L	Precentral Gyrus	4	-40	-14	52	0.0242	
7	L	Superior Temporal Gyrus	---	-62	-22	4	0.0360	2312
	L	Superior Temporal Gyrus	22	-52	-20	2	0.0328	
8	R	Precentral Gyrus	6	58	2	40	0.0448	2120
9	L	PHG (Amygdala)	---	-20	-4	-16	0.0553	2008
10	L	Superior Temporal Gyrus	22	-62	-38	18	0.0363	1896
11	R	Anterior Cingulate (MPFC)	---	2	40	-6	0.0299	1752
	L	Anterior Cingulate (MPFC)	---	-4	48	-8	0.0281	
12	R	Superior Temporal Gyrus	22	64	-28	4	0.0504	1608
13	R	Superior Temporal Gyrus	22	54	-16	6	0.0332	1352
	R	Superior Temporal Gyrus	22	62	-10	6	0.0266	

BA, Brodmann Area; MNI, Montreal Neurological Institute; ALE, activation likelihood estimation; L, Left; R, Right; BE-RBN, brand equity related brain network; DMN, default mode network; DMPFC, dorsal medial prefrontal cortex; MPFC, medial prefrontal cortex; PHG, parahippocampal gyrus; VS, ventral striatum.

Significant activated brain regions were divided into 13 clusters. Brain regions with the highest ALE value within each cluster are as follows: caudate (caudate body, Cluster1), claustrum (Cluster2), superior frontal gyrus (SFG, dorsal medial prefrontal cortex <DMPFC>, BA6, Cluster3), PHG (amygdala, Cluster4), MFG (MPFC, BA9, Cluster5), precentral gyrus (PreCG, BA4, Cluster6), superior temporal gyrus (STG, Cluster7), precentral gyrus (PreCG, BA6, Cluster7), PHG (amygdala, Cluster8), superior temporal gyrus (STG, BA22, Cluster9), superior temporal gyrus (STG, BA22, Cluster10), rACC (VMPFC, Cluster11), superior temporal gyrus (STG, BA22, Cluster11), and superior temporal gyrus (STG, BA22, Cluster12). Interestingly, even though the posterior regions were set as the ROIs, these regions disappeared. However, when setting loose thresholding criteria (uncorrected p < 0.01), these regions appeared. There is a concern about publication bias in these regions because of the FSN analysis results. Thus, the ALE map created by the MACM can be considered robust because brain regions derived from clusters with publication biases were eliminated. The result is partially consistent with results of previous subjective value-based decision-making studies even though the activations of the PCC were observed in a few of them (Acikalin et al., 2017; Bartra et al., 2013; Clithero and Rangel, 2014; Sescousse et al., 2013). This means that connections between the posterior region and other brain regions might be weak in the BE-RBN in comparison with a few subjective value-based decision making. The activations of the PCC seem to depend on types of reward modalities and decision-making modes (Acikalin et al., 2017; Bartra et al., 2013; Clithero and Rangel, 2014; Sescousse et al., 2013). Therefore, along with an activated position of the VMPFC, investigating the activation of the PCC is a useful cue for assessing reward modalities of brand equity.

### 3.3 Conjunction and contrast analysis

#### 3.3.1 Conjunction analysis

**Figure 4:**
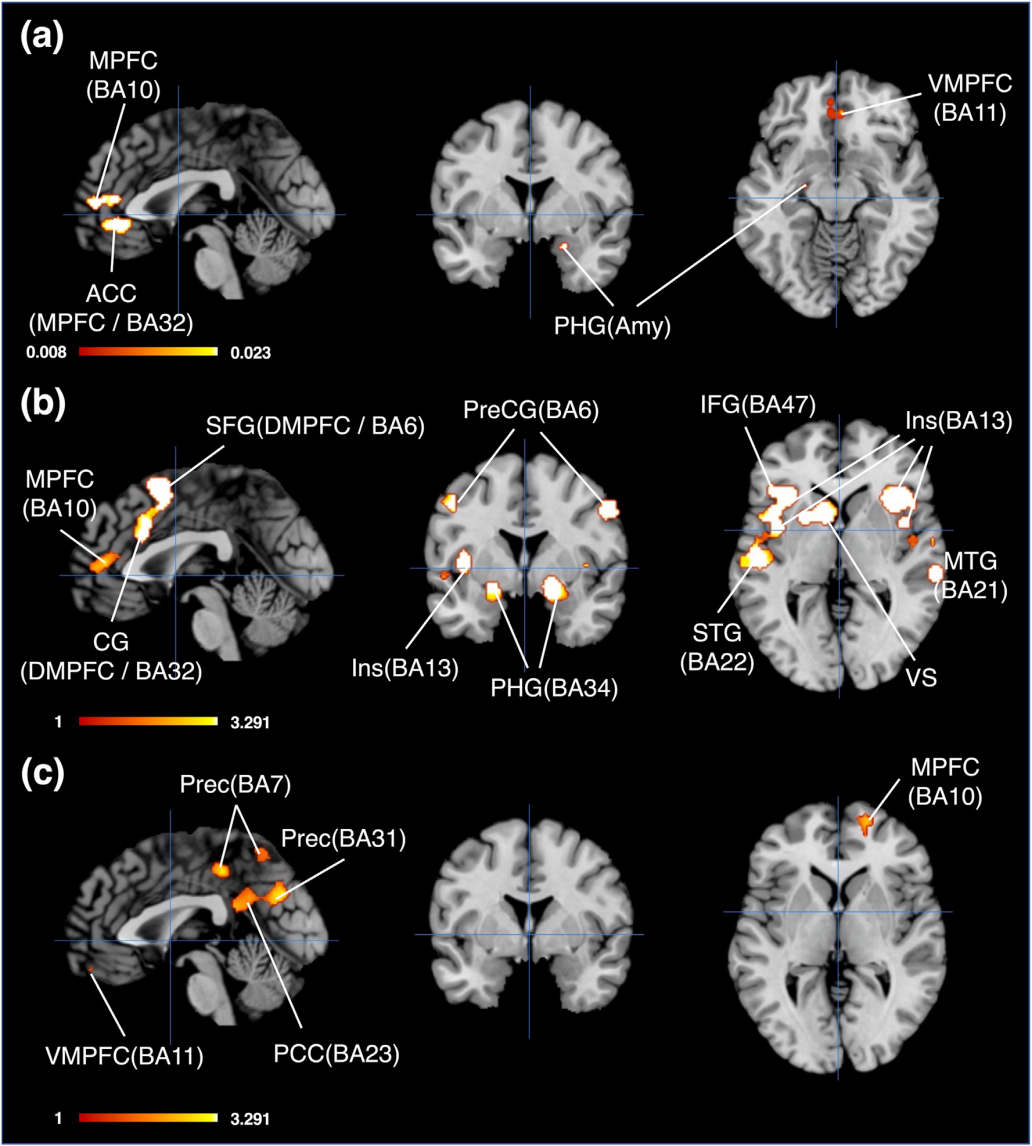
Results of conjunction and contrast analysis. (a)Results of conjunction analysis, Sagittal and Coronal view, Crosshairs (0, 0, 0); Axial view, Crosshair (0, -20, -13), (b) Results of contrast analysis (BE-RBN>DMN), Crosshairs (0, 0, 0), (c) Results of contrast analysis (BE-RBN<DMN), Crosshairs (0, 0, 0)/**Abbreviations**; ACC, anterior cingulate cortex; Amy, amygdala; BA, Brodmann area; BE-RBN, brand equity related brain network; CG, cingulate cortex; DMN, default, mode network; DMPFC, dorsal medial prefrontal cortex; HP, hippocampus; Ins, insula; MPFC, medial prefrontal cortex; MTG, middle temporal gyrus; PCC, posterior cingulate cortex; PHG, parahippocampal gyrus; PreC, precuneus; PreCG, precentral gyrus; SFG, superior frontal gyrus; STG, superior temporal gyrus; VMPFC, ventral medial prefrontal cortex; VS, ventral striatum.

The ALE map produced by conjunction analysis revealed five clusters in overlapping brain regions across both the BE-RBN and DMN (Table 4; Figure 4(a)): the rACC (cluster 1), MPFC (cluster 2), PHG (cluster 3, 4), and caudate (caudate head, VS; cluster 5). In particular, brain regions within the PHG were activated across cluster3 and cluster4. These regions were composed of the amygdala and entorhinal cortex (BA28, BA34). Therefore, the anterior part of the medial prefrontal region, the medial temporal region, and the ventral striatum were revealed as significantly characteristic brain regions shared between the BE-RBN and DMN. This result is almost the same as that found by Acikalin et al. (2017), excluding the PCC.

**Table 4:** The results of conjunction analysis (BE-RBN & DMN).

Cluster #	Cluster centered Coordinates (MNI)	Cluster Size (mm^3^)	Brain regions in the cluster (Gyrus/Cell type)	Peak voxel coordinates (MNI)			Side	Brain region	BA	ALE values
				x	y	z				
1	(-1, 41, -8)	1752	ACC/BA32, BA24	2	40	-6	R	ACC (MPFC)	---	0.0299
				-4	48	-8	L	ACC (MPFC)	---	0.0281
2	(-0, 50, 9)	1616	MFG, ACC/BA9, BA10, BA32,BA24	-4	54	8	L	MFG (MPFC)	9	0.0304
				4	40	10	R	ACC (MPFC)	32	0.0291
3	(-21, -8, -19)	320	PHG/Amy,BA34,BA28	-22	-10	-18	L	PHG (Amygdala)	---	0.0278
4	(24, -5, -21)	288	PHG/Amy,BA34	24	-4	-22	R	PHG (Amygdala)	---	0.0298
5	(-5, 9, -6)	24	CD/CH	-6	8	-6	L	Caudate Head (VS)	---	0.0232

BA, Brodmann Area; MNI, Montreal Neurological Institute; ALE, activation likelihood estimation; L, Left; R, Right; BE-RBN, brand equity related brain network; DMN, default mode network; ACC, anterior cingulate cortex; Amy, amygdala; CH, caudate head; MFG, medial frontal gyrus; MPFC, medial prefrontal cortex; PHG, parahippocampal gyrus; VMPFC, ventral medial prefrontal cortex; VS, ventral striatum.

#### 3.3.2 Contrast analysis

Distinctive brain regions of the BE-RBN were divided into 13 clusters (Table 5; Figure 4(b)). The brain regions revealed in each cluster are as follows: claustrum (Cluster1), insula (Cluster2), SFG (DMPFC, BA6, Cluster3), lentiform nucleus (medial globus pallidus <MGP>, Cluster4), MFG (MFPC, BA9, Cluster5), precentral gyrus (PreCG, BA4, Cluster6), precentral gyrus (PreCG, BA6, Cluster7), precentral gyrus (PreCG, BA6, Cluster8), lentiform nucleus (lateral globus pallidus <LGP>, Cluster9), superior temporal gyrus (STG, BA22, Cluster10), superior temporal gyrus (STG, BA22, Cluster11), postcentral gyrus (PoCG, BA40, Cluster12), and rACC (BA32, VMPFC, Cluster13). Cluster1 broadly covers brain regions including the insula, striatum, and lateral cortical regions such as the STG, inferior frontal gyrus, and PreCG. Both cluster4 and cluster8 cover the VS, the bilateral PHG, including the amygdala and entorhinal cortex (BA28, BA34). Although the MPFC corresponds to cluster3, cluster5, and cluster13, the positions of these clusters are different. Cluster3 is placed at the dorsal MPFC, while both cluster5 and cluster13 are placed at the ventral MPFC. Clusters 2, 3, 6, 7, 9, 10, 11, and 12 broadly cover the lateral parts of the cortical regions, excluding the posterior parts. Interestingly, the insula is included in clusters 1, 2, 9, and 10. The anterior insula corresponds to clusters 1 and 2, while the posterior insula corresponds to clusters 9 and 10. Moreover, although the PHG and the VS were observed in overlapping regions between the BE-RBN and DMN, these regions were also detected in this contrast analysis. This means that these regions might have more intensive and varied commitments to functions of the BE-RBN than the DMN. Although Acikalin et al. (2017) demonstrated that the MPFC was characteristically activated in the more anterior part of the subjective value network than in the DMN, this study showed that the activated MPFC was observed in the posterior parts in comparison with the regions observed in the DMN.

**Table 5:** The result of the contrast analysis (BE-RBN > DMN).

Cluster #	Cluster centered Coordinates (MNI)	Cluster Size (mm^3^)	Brain regions Included in Cluster (Gyrus/Cell type)	Peak voxel coordinates (MNI)			Side	Brain region	BA
				x	y	z			
1	(-36, 7, 1)	13608	Ins,CD,LN,STG,IFG,Cla,PreCG/BA13,BA22,BA44,BA45,BA47,CH,CB,Put	-35	8	2	L	Claustrum	---
				-65	-20	2	L	STG	---
				-52	2	-6	L	STG	22
2	(41, 17, -1)	5760	Ins,Cla,IFG,PreCG,STG,ExN/BA13,BA22,BA44,BA45,BA47	41	17	-2	R	Insula	---
3	(0, 13, 48)	5456	MFG,CG, SFG/BA6,BA32,BA24	0	12	49	L	SFG (DMPFC)	6
				-1	17	43	L	MFG (DMPFC)	6
4	(20, -5, -14)	3384	LN,PHG/MGP,Amy,LGP, BA34,BA28,Put	19	-5	-13	R	MGP	---
5	(-2, 48, 10)	3144		-6	50	8	L	MFG (MPFC)	9
			ACC,MFG/BA32,BA9,BA10, BA24	-6	44	10	L	ACC (MPFC)	32
				6	39	15	R	ACC (MPFC)	32
6	(-47, -7, 46)	2808	PreCG, PoCG/BA4,BA6,BA3	-46	-7	46	L	Precentral gyrus	4
				-44	4	36	L	Precentral gyrus	6
7	(55, -1, 38)	2120	PreCG, MidFG/BA6,BA4	55	-1	38	R	Precentral gyrus	6
8	(-20, -5, -14)	2096	PHG,LN/Amy,LGP,MGP, BA34,BA28,Put	-20	-5	-13	L	LGP	---
				-21	-3	-20	L	PHG (Amygdala)	---
9	(-61, -37, 15)	2016	STG,MidTG,Ins,IPL,PoCG/BA22,BA13,BA42,BA40	-61	-37	14	L	STG	22
10	(56, -13, 5)	1976	Ins,STG,TTG,PreCG/BA13, BA22,BA41,BA42,BA43	62	-10	6	R	STG	22
				51	-14	5	R	Insula	13
				50	-22	6	R	STG	13
				50	-10	-2	R	Insula	13
				50	-4	-2	R	Insula	13
11	(63, -29, 4)	1664	MidTG,STG/BA22,BA21, BA42,BA41	63	-30	4	R	STG	22
12	(64, -25, 23)	224	PoCG,IPL/BA40	66	-24	22	R	Postcentral gyrus	40
13	(-7, 38, -12)	104	ACC/BA32	-8	38	-12	L	ACC (VMPFC)	32

BA, Brodmann Area; MNI, Montreal Neurological Institute; ALE, activation likelihood estimation; L, Left; R, Right; BE-RBN, brand equity related brain network; DMN, default mode network; ACC, anterior cingulate cortex; Amy, amygdala; CD, caudate; CG, cingulate gyrus; Cla, claustrum; DMPFC, dorsal medial prefrontal cortex; ExN, extra-nuclear; IFG, inferior frontal gyrus; IPL, inferior parietal lobule; Ins, insula; LGP, lateral globus pallidus; LN, lentiform nucleus; MFG, medial frontal gyrus; MGP, medial globus pallidus; MidFG, middle frontal gyrus; PHG, parahippocampal gyrus; PoCG, postcentral gyrus; PreCG, precentral gyrus; Put, putamen; SFG, superior frontal gyrus; STG, superior temporal gyrus; TTG, transverse temporal gyrus; VMPFC, ventral medial prefrontal cortex.

For the DMN, distinct brain regions were divided into eight clusters (Table 6; Figure 4(c)). The brain regions revealed in each cluster are as follows: middle cingulate cortex (MCC, BA31, cluster 1), middle temporal gyrus (MTG, BA39, cluster 2), SFG (BA8, cluster 3), medial frontal gyrus (MFG, MPFC, cluster 4), medial frontal gyrus (MFG, MPFC, BA10, cluster 5), subcallosal gyrus (MFG, MPFC, BA25, cluster 6), medial frontal gyrus (MFG, MPFC, BA11, cluster 7), and middle temporal gyrus (MTG, BA39, cluster 8). Cluster1 comprised brain regions covering the medial to lateral areas of the posterior cortical region. Most of the brain regions of the PCC were included in cluster1. The MPFC corresponded to clusters 4, 5, 6, and 7. In clusters 4 and 5, the anterior parts of the MPFC were activated. In clusters 5 and 6, subgenual parts of the MPFC were activated. Thus, for the DMN, characteristically activated brain regions were broadly distributed in the MPFC and PCC. These regions have been known as the core modules composing the DMN core network (Andrews-Hanna, 2012). The MPFC is the module of the anterior DMN, while the PCC is the module of the posterior DMN.

**Table 6:** The result of the contrast analysis (BE-RBN < DMN).

Cluster #	Cluster centered Coordinates (MNI)	Cluster Size (mm^3^)	Brain regions Included in Cluster (Gyrus/Cell type)	Peak voxel coordinates (MNI)			Side	Brain region	BA
				x	y	z			
1	(-2, -54, 36)	9184	Prec,CG,PCC,Cun,ParaCL/BA31,BA7,BA30,BA18,BA23	-5	-35	45	L	Cingulate gyrus	31
				-5	-72	31	L	Precuneus	31
				-8	-58	33	L	Precuneus	31
				2	-46	22	L	PCC	29
				0	-42	22	L	PCC	30
				2	-51	31	L	Cingulate gyrus	29
				10	-46	32	R	Precuneus	31
				-6	-58	46	L	Precuneus	7
				1	-55	42	L	Precuneus	7
				9	-59	44	R	Precuneus	7
				12	-54	32	R	Cingulate gyrus	31
2	(49, -69, 25)	2152	MidTG,AG,STG,Prec/BA39,BA19	48	-71	27	R	MidTG	39
3	(-25, 21, 45)	912	MidFG,SFG/BA8,BA6	-21	19	45	L	SFG	8
4	(17, 59, -1)	640	MFG/BA10	20	62	-2	R	MFG	10
				18	58	-2	R	MFG	10
5	(6, 52, -17)	560	MFG,ACC/BA10,BA11,BA32	6	52	-18	R	MFG (VMPFC)	10
				10	54	-11	R	MFG (VMPFC)	10
6	(5, 13, -18)	224	SubCG,ACC/BA25	3	11	-18	R	Subcallosal gyrus	25
7	(8, 33, -19)	200	MFG,ACC/BA11,BA32	8	34	-22	R	MFG (VMPFC)	11
				8	32	-18	R	ACC (VMPFC)	32
8	(-44, -72, 20)	184	MidTG/BA39	-44	-74	20	L	MidTG	39
				-44	-70	16	L	MidTG	39

BA, Brodmann Area; MNI, Montreal Neurological Institute; ALE, activation likelihood estimation; L, Left; R, Right; ACC, anterior cingulate cortex; AG, angular gyrus; CG, cingulate gyrus; Cun, cuneus; MFG, medial frontal gyrus; MidFG, middle frontal gyrus; MidTG, middle temporal gyrus; PCC, posterior central cortex; ParaCL, Paracentral Lobule; Prec, precuneus; SFG, superior frontal gyrus; STG, Superior temporal gyrus; SubCG, subcallosal gyrus; VMPFC, ventral medial prefrontal corte.

### 3.4 Decoding analysis

**Figure 5:**
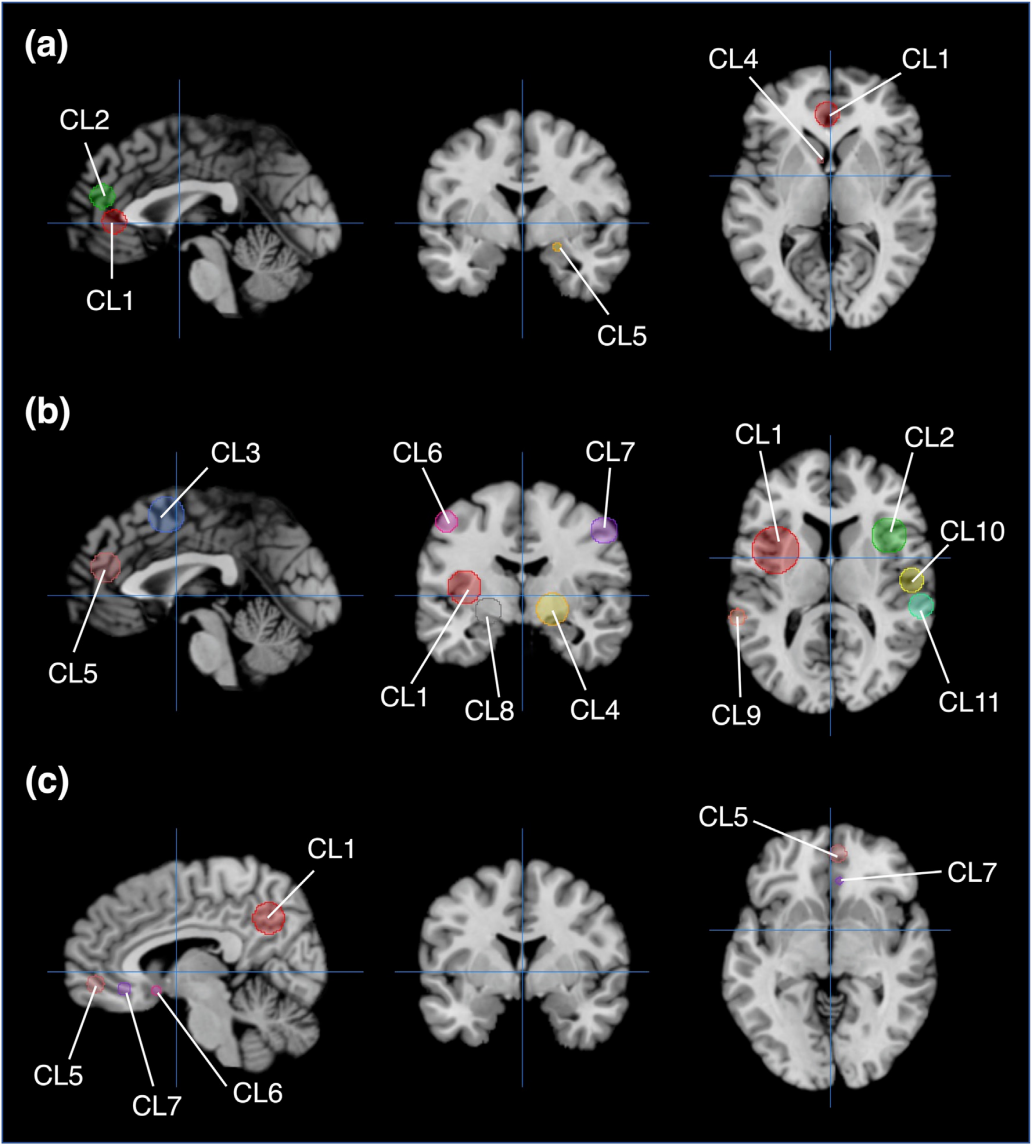
Spherical ROIs created based on the results of conjunction and contrast analysis. (a)Results of conjunction analysis, Sagittal and Axial view, Crosshairs (0, 0, 0); Coronal view, Crosshair (0, -2, 0), (b) Results of contrast analysis (BE-RBN>DMN), Sagittal view, Crosshairs (0, 0, 0); Coronal view, Crosshairs (0, -6, 0); Axial view, Crosshairs (0, 0, 7) (c) Results of contrast analysis (BE-RBN<DMN), Sagittal view, Crosshairs (6, 0, 0); Coronal view, Crosshairs (0, 0, 0); Axial view, Crosshairs (6, 0, -7)/**Abbreviations**; CL, cluster.

To decode the revealed distinctive and shared brain regions between the BE-RBN and the DMN using the behavioral analysis plugin tool, we created the ROIs based on the Talairach coordinate template using Mango software. The Talairach template was created by modifying the MNI template with the transform function in Mango software. All ROIs were created according to brain region clusters revealed by the conjunction and contrast analysis, and spherical ROIs were constructed. We set each radius of these ROIs to match the actual clusters’ volume size. For example, the volume of cluster 1 in the result of conjunction analysis was 1752 mm^3^, and the radius was set as 7.478mm. The radius was set similarly for other clusters as well. We adopted the center coordinate in each cluster as the focus of each ROI. Center coordinates based on the MNI space were transformed to the Talairach space. The set ROIs are shown in Figure 5.

In brain regions shared between the BE-RBN and DMN, characteristic behavior and cognitive function are shown in Table 7. Significant domains and sub-domains in behavior and cognitive function were observed in only two clusters (cluster 1 and 3). In cluster 1, “Emotion, Positive (Reward/Gain)” and “Cognition, Reasoning” were significant. In cluster3, “Cognition, Memory (Explicit)” and “Perception, Vision (Unspecified)” were significant.

**Table 7:** The results of the decoding analysis.

Analysis	Cluster	Domain	Sub-domain	Z-score
BE-RBN&DMN	Cluster1	Emotion	Positive (Reward/Gain)	3.641
		Cognition	Reasoning	3.315
	Cluster3	Cognition	Memory (Explicit)	3.162
		Perception	Vision (Unspecified)	3.109
BE-RBN>DMN	Cluster1	Perception	Somesthesis (Pain)	9.884
		Cognition	Attention	8.733
		Action	Execution (Unspecified)	7.502
		Cognition	Language (Speech)	7.198
		Cognition	Language (Semantics)	5.878
		Action	Execution (Speech)	5.725
		Emotion	Positive (Reward/Gain)	5.558
		Action	Inhibition	5.011
		Perception	Gustation	4.341
		Cognition	Music	4.262
		Interoception	Sexuality	3.769
		Cognition	Memory (Explicit)	3.758
		Perception	Somesthesis (Unspecified)	3.716
		Interoception	Thermoregulation	3.314
		Perception	Olfaction	3.179
		Cognition	Reasoning	3.172
		Emotion	Positive (Happiness)	3.007
	Cluster2	Cognition	Attention	10.332
		Perception	Somesthesis (Pain)	9.806
		Emotion	Positive (Reward/Gain)	7.229
		Cognition	Reasoning	6.700
		Action	Inhibition	6.660
		Cognition	Language (Semantics)	5.420
		Cognition	Language (Speech)	5.326
		Perception	Audition	5.212
		Cognition	Memory (Working)	5.081
		Cognition	Music	5.002
		Cognition	Memory (Explicit)	4.952
		Perception	Gustation	4.304
		Interoception	Thermoregulation	4.113
		Action	Execution (Unspecified)	4.048
		Emotion	Negative (Sadness)	3.924
		Cognition	Language (Phonology)	3.515
		Perception	Somesthesis (Unspecified)	3.491
		Emotion	Negative (Fear)	3.479
		Cognition	Spatial	3.478
		Action	Execution (Speech)	3.382
		Interoception	Sexuality	3.276
		Emotion	Negative (Unspecified)	3.013
		Emotion	Negative (Anxiety)	3.011
	Cluster3	Cognition	Attention	13.350
		Action	Execution (Unspecified)	12.635
		Cognition	Memory (Working)	10.558
		Cognition	Language (Semantics)	10.215
		Cognition	Language (Speech)	9.959
		Perception	Somesthesis (Pain)	8.202
		Cognition	Reasoning	7.373
		Cognition	Memory (Explicit)	6.892
		Action	Execution (Speech)	6.827
		Action	Inhibition	6.799
		Perception	Vision (Motion)	6.540
		Perception	Audition	6.309
		Cognition	Language (Phonology)	5.785
		Cognition	Music	5.590
		Perception	Somesthesis (Unspecified)	5.432
		Perception	Vision (Shape)	5.207
		Action	Imagination	4.868
		Emotion	Positive (Reward/Gain)	4.836
		Cognition	Language (Orthography)	4.632
		Perception	Vision (Unspecified)	4.572
		Emotion	Negative (Unspecified)	4.295
		Cognition	Spatial	3.896
		Cognition	Social Cognition	3.304
	Cluster4	Emotion	Negative (Unspecified)	4.694
		Emotion	Negative (Fear)	4.649
		Perception	Olfaction	4.438
		Interoception	Sexuality	3.946
		Emotion	Negative (Sadness)	3.810
		Perception	Vision (Unspecified)	3.540
		Emotion	Positive (Unspecified)	3.510
		Emotion	Negative (Disgust)	3.368
		Emotion	Positive (Reward/Gain)	3.051
		Emotion	Negative (Anger)	3.017
	Cluster6	Cognition	Language (Speech)	6.881
		Action	Execution (Unspecified)	6.269
		Action	Execution (Speech)	5.308
		Cognition	Language (Semantics)	4.319
		Cognition	Memory (Working)	4.090
		Cognition	Music	3.736
		Perception	Vision (Motion)	3.680
		Cognition	Attention	3.301
		Perception	Somesthesis (Unspecified)	3.147
	Cluster7	Action	Execution (Unspecified)	6.305
		Action	Execution (Speech)	5.777
		Cognition	Language (Speech)	4.020
		Perception	Vision (Motion)	3.293
		Perception	Audition	3.086
	Cluster8	Emotion	Negative (Fear)	5.630
		Perception	Vision (Unspecified)	5.094
		Emotion	Negative (Unspecified)	4.984
		Perception	Olfaction	4.847
		Emotion	Negative (Sadness)	4.329
		Emotion	Positive (Reward/Gain)	4.152
		Emotion	Negative (Disgust)	4.032
		Emotion	Positive (Happiness)	4.010
		Cognition	Attention	3.950
		Interoception	Sexuality	3.911
		Cognition	Reasoning	3.886
		Cognition	Memory (Unspecified)	3.675
		Emotion	Positive (Unspecified)	3.582
		Perception	Vision (Shape)	3.421
		Cognition	Memory (Expliicit)	3.084
	Cluster9	Perception	Audition	6.181
		Cognition	Music	4.561
		Cognition	Language (Speech)	4.233
	Cluster10	Perception	Audition	5.852
		Cognition	Music	4.666
		Action	Execution (Speech)	4.538
		Cognition	Language (Speech)	3.578
	Cluster11	Perception	Audition	6.095
		Cognition	Language (Speech)	4.668
		Cognition	Music	4.198
		Action	Execution (Speech)	3.925
BE-RBN<DMN	Cluster1	Cognition	Social Cognition	6.240
		Cognition	Memory (Explicit)	3.350

Only sub-domains with z-score 3.0 or more were listed; BE-RBN, brand equity related brain network; DMN, default mode network.

In significantly activated brain regions in the BE-RBN (BE-RBN > DMN), significantly characteristic behavior and cognitive function taxonomy were observed in nine clusters (clusters 1, 2, 3, 4, 6, 7, 8, 9, 10, and 11). Detailed results are shown in Table 7. Multiple domains (four domains or more were included) were listed in clusters 1, 2, 3, and 8. The “Emotional” domain was dominant in cluster 4, while the “Cognitive” domain occupied cluster 6. The “Perception” domain was included in all clusters where significant sub-domains were observed. Regarding the sub-domains, the “Positive (Reward/Gain)” was listed in all clusters that included the “Emotional” domain. On the other hand, the “Language” related sub-domain was listed in all clusters that included the “Cognitive” domain. In particular, “Language (Speech)” covered all clusters that included the “Cognitive” domain. “Language (Semantic)” was included in four clusters (clusters 1, 2, 3, and 6). In the “Cognitive” domain, other characteristic sub-domains were “Attention”, “Memory (Explicit)”, and “Reasoning”. “Attention” was included in five clusters (clusters 1, 2, 3, 6, and 8), and both “Memory (Explicit)” and “Reasoning” were included in four clusters (clusters 1, 2, 3, and 8). Interestingly, although the “Interoception” domain has 11 sub-domains, only two sub-domains (“Sexuality” and “Thermoregulation”) were significant. In particular, “Sexuality” was listed in all clusters that included the “Interoception” domain (clusters 1, 2, 4, and 8). Regarding the sub-domains of “Perception”, any of those related to the five senses (“Audition”, “Gustation”, “Olfaction”, “Somesthesis”, and “Vision”) were listed in each cluster that included significant sub-domains. Regarding the sub-domains of “Action”, four sub-domains (“Execution [Speech]”, “Execution [Unspecified]”, “Imagination” and “Inhibition”) were significant. “Execution (Speech)” was listed in all clusters that included the “Action” domain (clusters 1, 2, 3, 6, 7, and 10).

In significantly activated brain regions in the DMN (BE-RBN < DMN), significant sub-domains were observed only in cluster 1: “Cognition, Social cognition” and “Cognition, Memory (Explicit)”.

### 3.5 FSN analysis

The cluster-wise data on FSN is shown in Supplementary Table S2. The number of FSN in clusters 1–3 highly exceeded the minimum FSN. Especially regarding clusters 1 and 2, the FSN analysis was stopped at the maximum FSN because the cluster was significant at even the maximum FSN. The FSN analysis in these clusters proved that the ALE results on brand related brain regions were robust against potential publication bias. However, the “file drawer problem” was confirmed in cluster 4 and 5 because the number of FSN in these clusters were smaller than the minimum FSN. Hence, robustness of the ALE results in these clusters is considered to be low.

## 4 Discussion

Broad, overlapping regions were observed in the MPFC. Especially, as shown in the decoding analysis, the VMPFC plays a crucial role in computing and integrating subjectively experienced reward values derived from different kinds of reward stimuli (Sescousse et al., 2013), including ratings of pleasantness (Peters and Büchel, 2010), tracking of the value of financial payoffs (O'Doherty et al., 2001), and emotional processes (Bush et al., 2000). The amygdala is associated with emotional processing (LeDoux, 1992; Sergerie et al., 2008). However, the “Emotional” domain was not significant in the decoding analysis. The VS is associated with reward processing. The VS encodes expected reward values and negative values, such as punishments (Delgado et al., 2000). Its anterior parts are associated with positive rewards such as euphoria (Knutson et al., 2001). The VS also plays a key role in reward-related approaches and avoidance behaviors (Haber and Knutson, 2010). Motoki et al. (2019) reported that the VS is the core region that represents the values of utilitarian and hedonistic goods. A large number of studies has shown correlations between the VS and rewards. Activities in this area are associated with the magnitude of cumulative rewards (Elliott et al., 2000), anticipation of reward (Knutson et al., 2001), and social rewards such as evaluation of faces (Aharon et al., 2001), reputation, and social hierarchy (Izuma et al., 2008). Thus, the VS is thought to compute reward values, which are prediction errors between expected rewards and outcomes (Niv and Schoenbaum, 2008). Moreover, the VMPFC and the VS were crucial modules of the neural common currency (NCC) network (Bartra et al., 2013; Levy and Glimcher, 2012). These brain areas are dopaminergic midbrain regions and are known as the core regions of the NCC hypothesis. In the NCC hypothesis, subjective values are thought to be encoded in the same brain regions by a single scale, regardless of reward type. Many empirical studies have proven this hypothesis (Bartra et al., 2013). Activities in these areas are associated with various types of subjective reward magnitude (Bartra et al., 2013), the subjective valuations of gains and losses in behavioral loss aversion (Tom et al., 2007), discount function, subjective values of delayed monetary rewards (Kable and Glimcher, 2007), and outcome values of rewards (Bartra et al., 2013). Given that the decoded results showed that “Positive (Reward/Gain)” was significant in these regions, it can be considered that these regions are also associated with reward processing. Although, both the VS and amygdala are considered crucial modules of reward networks (Camara et al., 2009; Seitzman et al., 2020), “Positive (Reward/Gain)” was not significant in clusters 4 and 5. The VMPFC and entorhinal cortex are both core components of the medial temporal lobule (MTL) subsystem in the DMN. The MTL subsystem is involved in episodic and autobiographical memory (Andrews-Hanna, 2012). The decoded result shows that “Cognition, Memory (Explicit)” was significant in cluster 3. Thus, the overlapping brain regions can be considered to be functions of self-referential, reward, memory processing.

Although the PHG (the amygdala and entorhinal cortex <BA28, BA34>) overlapped with the BE-RBN and DMN, broadly strong activations in these regions were also observed in the brain regions of “the BE-RBN > the DMN”. Interactions between the amygdala and entorhinal cortex in the PHG were engaged in the formation of emotional episodic memory (Kesner and Rogers, 2004; Phelps, 2004), emotional autobiographical memory (Buchanan et al., 2006) and associative memory related to episodic memories (Aminoff et al., 2013). These considerations are consistent with the decoded results. Regarding these regions that correspond with cluster 4 and 8 (BE-RBN > DMN) in the decoding analysis, both many sub-domains of the “Emotional” and “Memory (Explicit)” sub-domains were significant in cluster 8. Additionally, in these clusters, “Perception” (sub-domain; “Vision” and “Olfaction”) was also significant. The PHG is involved in associative memories related to visuospatial and odor information (Aminoff et al., 2013; Dahmani et al., 2018; Zhou et al., 2021). Willander and Larsson (2006) reported that autobiographical memories related to odor information was more vividly recalled than other sensory information (Willander and Larsson, 2006).

In contrast, the contrast analysis (BE-RBN > DMN) result showed that the insula covered four clusters (clusters 1, 2, 9, and 10). This indicates that the insula can be considered a distinct brand equity-related brain region. The insula is engaged in various emotional and cognitive mental processes derived from self-generated interoceptive feelings (Craig, 2003; Damasio et al., 2000; Naqvi and Bechara, 2010). Since the anterior insula is the center of the salience network, it has connections with the amygdala and VS (Menon, 2015; Menon and Uddin, 2010; Seeley et al., 2007).

The interconnections among these brain regions are associated with behaviors driven by impulsive feelings, such as addiction, sexual emotion, and drug abuse (Childress et al., 2008; Gola et al., 2015). Actually, the “Sexuality” sub-domain in the “Interoception” domain was significant in clusters that demonstrated these connections (i.e., clusters 1, 2, 4 and 8 [BE-RBN > DMN]). As shown in the decoded result that “Positive (Reward/Gain)” was significant in clusters 1 and 2, activations in the anterior insula were observed in the reward and value-based decision-making contexts (Acikalin et al., 2017; Bartra et al., 2013; Brown et al., 2011; Sescousse et al., 2013). Prueschoff et al. (2008) reported that the anterior insula is engaged in risk learning in risky decision making (Preuschoff et al., 2008). In addition, the posterior insula has connections with the sensorimotor cortex and the parietal cortex (i.e., the precentral gyrus and the postcentral gyrus; Taylor et al., 2009a, Taylor et al., 2009b; Uddin et al., 2017). These regions play a crucial role in sensorimotor processing (Uddin et al., 2017). Similar to the anterior insula, the posterior insula is also involved in visceral processing such as sexual emotion (Cera et al., 2020). These considerations were partly proven in the decoding analysis. The “perception” domain was significant in clusters that demonstrated an interconnection among the posterior insula, sensorimotor cortex, and parietal cortex (i.e., clusters 6, 7, 9, and 10). However, the “Sexuality” sub-domain in the “Interoception” domain was not significant in these clusters. Additionally, the results for clusters 1, 2, and 3 showed that semantic memory-related sub-domains such as “Language (Semantic)” and “Cognition, Memory (Explicit)” were significant. The IFG (clusters 1,2) and SFG (DMPFC, cluster 3) included in these clusters are both associated with semantic memory (Binder et al., 2009), implying its influence on brand equity-related mental processes. Connections of the DMPFC, IFG, and PHG are involved in semantic associative memory (Addis and McAndrews, 2006). This interpretation is consistent with the findings of previous consumer neuroscience studies (Chen et al., 2015; McClure et al., 2004; Yoon et al., 2006). Interconnections within the DMPFC, the IFG and the PHG are associated with familiarity, along with various sensory modalities such as visual and music (Satoh et al., 2006; Taylor et al., 2009a, Taylor et al., 2009b). Taking into consideration the discussion on episodic and autobiographical memory as described above, memory processing plays a crucial role in brand equity-related mental processes. In particular, the comprehensive associative memory system, which includes semantic, episodic and autobiographical memories, may be intensively involved in brand association facet, which is a component of brand equity. Brand associations is a construct of combining brand name with brand knowledge, including multiple modalities derived from marketing communications and other experiences between brands and consumers (Aaker, 1992; Keller, 2016). Constructing a comprehensive associative memory system might generate a sense of familiarity toward brands. Therefore, brand association can be considered as a construct centrally functioned by the PHG. Moreover, given that activations of the PHG, which is located primarily in the entorhinal cortex <BA28, BA34>, were not reported in previous subjective value-based decision-making studies (Acikalin et al., 2017; Bartra et al., 2013; Clithero and Rangel, 2014; Sescousse et al., 2013), the associative memory system driven by the PHG can be presumed to be a distinct, characteristic mental process among various value-based decision-making processes. When combined, brain regions in “BE-RBN > DMN” are considered to play a role in self-referential, reward, emotional, memory and sensorimotor processing.

In contrast, the contrast analysis (BE-RBN < DMN) revealed that the anterior areas of both the MPFC (aMPFC) and PCC are the main distinctive brain regions in the DMN. The aMPFC and PCC are both crucial core nodes in the various types of DMN subsystem studies (Andrews-Hanna, 2012; Damoiseaux et al., 2008). The aMPFC is the core node of the anterior DMN (Damoiseaux et al., 2008). The PCC is the core node of the posterior DMN (Damoiseaux et al., 2008). The aMPFC is involved in the shifting of attention to future directions, the imaging of future results (Addis et al., 2007), and imaging future outcomes (Gerlach et al., 2014). The increased activations in the PCC were observed when simulating the future and when reflecting on the future self (Stawarczyk and D'Argembeau, 2015; Xu et al., 2016). This region is also involved in social cognitions such as simulating others' mental state and theory of mind (Esménio et al., 2020; Mars et al., 2012). In addition, the retrosplenial regions of the PCC have connections with the MTL, the region associated with episodic and autobiographical memory (Andrews-Hanna, 2012; Xu et al., 2016). Although these mental processes were not significant regarding the aMPFC, both the “Social cognition” and “Memory (Explicit)” sub-domains were significant in the PCC. Compared with the BE-RBN, given that the PCC might be the strongly distinctive brain region, social cognition-related mental processes can be considered as characteristic in the DMN. While activations of the PCC were observed in other value-based decision-making studies (Acikalin et al., 2017; Bartra et al., 2013; Clithero and Rangel, 2014; Sescousse et al., 2013), no activations were observed in the BE-RBN. Thus, the brand equity related mental processes are presumed to be a construct with weak social cognitive aspects, although the “Social Cognition” sub-domain was significant in cluster 3 of the contrast analysis (BERBN > DMN). From the view of reward modalities, although activations of the PCC were observed in monetary rewards, little activations of the PCC were shown in primary (food, erotic) and aesthetic rewards (Bartra et al., 2013; Brown et al., 2011; Sescousse et al., 2013). Moreover, a few studies pointed out that abstract rewards, such as money, are encoded in the more anterior parts of the VMPFC (Clithero and Rangel, 2014; Sescousse et al., 2013). Conversely, primary rewards, which include foods and erotic stimuli, are encoded in the more posterior parts of the VMPFC (Clithero and Rangel, 2014; Sescousse et al., 2013). According to Brown et al. (2011), activations of the posterior part of the VMPFC were observed in aesthetic processing (Brown et al., 2011). The VMPFC's activated areas in the BE-RBN were located in its posterior parts. As a result of activated position of the VMPFC and deactivations of the PCC, a brand with brand equity can be considered a less abstract reward akin to primary and aesthetic rewards.

Therefore, while we revealed that the BE-RBN overlaps with the MPFC and the MTL sub-system, excluding the PCC retrosplenial regions, we also identified several distinctive brain regions of the BE-RBN apart from the DMN, such as the IFG, insula, VS and parietal regions. This result indicates that the function of the DMN alone might be insufficient to engage in brand equity-related mental processes. Moreover, the decoded results demonstrate that these brain regions are associated with self-referential processing, reward processing, emotional processing, memory processing and sensorimotor processing. Thus, brand equity-related mental processes can be considered as complex constructs composed of these multiple mental processes. Thus, although each mental process has been observed in previous consumer neuroscience studies (Chen et al., 2015; McClure et al., 2004; Reimann et al., 2011; Schaefer and Rotte, 2007; Yoon et al., 2006), brand equity-related mental processes can be considered complex constructs in which these multiple mental processes are integrated. In other words, they can be interpreted as subjective and rewards processing, involving an impulsive emotional associative memory system with multiple sensory modalities inputted. These findings imply that the DMN-like mental processes, such as spontaneous brand recall and self-expressive benefit reported in the marketing literature, can be a construct underlined by not only the DMN, but also other brain networks. The influence of social cognitive processing on the brand equity-related mental processes is considered to be marginal. For instance, self-expressive benefit allows brands to realize symbols of a person's self-concept (Aaker, 1996). A consumer wants to be perceived as a creative person by using Apple, or as a sophisticated and elegant person by using Lancome, regardless of the consumer's current status. When an individual watches advertising, sees a logo while walking in the street, or reads news on an SNS, their emotions about a brand would accumulate. Thus, consumers' motivation to purchase brands is to realize their aspirations (Cho et al., 2015). This accumulated desire to fulfill self-oriented objectives, along with their aspirations, can be attributed to subjective and reward processing with strong emotional associative memories.

Even though our study has its strengths and we showed that our ALE results, which were used as ROIs for the MACM, were robust by using FSN analysis, it also has a few limitations. First, although we revealed brain regions related to brand equity by aggregating studies using branded objects as stimuli, we did not consider studies using non-branded objects as stimuli. To specify characteristic brain regions in association with brand equity, it would be more desirable for us to compare brand regions related to brand equity with those related to non-branded objects. In our next study, we will perform this comparison. Moreover, we need to assess differentiations between branded objects and non-branded objects in terms of reward modalities. It is especially important to investigate variations on VMPFC positions between them. Regarding this issue, to more precisely identify distinct mental processes of brand equity-related value-based decision making, we need to compare these consumer contextual reward modalities with general reward modalities such as monetary, primary, and aesthetic modalities with rigorous methods. In addition, we noted that memory processing may be a characteristic mental process related to brand equity-related decision-making. However, to precisely identify its characteristic mental processes, BE-RBN needs to be directly compared to the other value-based decision-making brain networks. Second, we conducted an analysis using cross-sectional selected studies that covered various kinds of tasks, stimulus types, and product categories. While this approach has the advantage of being able to take universal outcomes, weak explanatory regions might emerge. For example, there might be differences in brain activated regions in brand equity between brands selling durable versus fast-moving consumer goods. Similarly, even if stimuli might be in the same product category, there may be different brain activated patterns depending on respondents’ profiles, such as age, gender, and personality. Additionally, as a minor point, although spherical ROIs set for decoding analysis were created to match the size of the actual clusters revealed by the ALE analysis, there are some errors in comparison to the size of the actual clusters. For this reason, the decoded results in some clusters might be of low precision. Our study is of great significance for brand equity studies. However, further research is needed.

## 5 Conclusion

We revealed that the BE-RBN is a neural mechanism mainly underlined by interactions among the DMPFC, VMPFC, IFG, insula, PHG, VS, and parietal regions. Given that each brain region is a component of a few brain networks (i.e., the DMN, the salience network, the NCC network and sensorimotor network), the BE-RBN can be considered as a neural mechanism interplayed by multiple brain networks. This implies that the DMN limitedly influences the brand equity-related mental processes. From the decoded results, we identified that the mental processes of brand equity are a complex construct that integrates multiple mental processing (i.e., self-referential, reward, emotion, memory, sensorimotor). Our study shows that a brand is not just a reward. For a brand in food category, it could be difficult to build brand equity by only providing good taste. To build brand equity, it might be insufficient to only communicate self-relevant content to consumers through digital media using a machine learning algorithm. Marketers need to conduct initiatives to stimulate consumers' senses and provide experiences to consumers from omnidirectional contact points. These stimuli and experiences must be comfortable, beneficial, and made from the activation of consumers' self-relevant memories. Therefore, marketers need to execute initiatives to integrate consumers' mental processes related while building brand equity. In addition, our results provide guidelines for measuring brand equity. Consequently, marketers need to set multiple variables covering the five mental processes of the consumer (i.e., self-referential, reward, emotion, memory, and sensorimotor) if they would like to comprehensively detect consumers’ perceptions regarding brand equity. Our findings may contribute to more accurate brand equity studies.

## Declarations

### Author contribution statement

Shinya Watanuki: Conceived and designed the experiments; Performed the experiments; Analyzed and interpreted the data; Contributed reagents, materials, analysis tools or data; Wrote the paper.

Hiroyuki Akama: Analyzed and interpreted the data; Contributed reagents, materials, analysis tools or data.

### Funding statement

Dr. SHINYA Watanuki was supported by 10.13039/501100001691Japan Society for the Promotion of Science [JP20K13633].

### Data availability statement

The datasets generated for this study are available on request to the corresponding author.

### Declaration of interest's statement

The authors declare no conflict of interest.

### Additional information

No additional information is available for this paper.

## Appendix A Supplementary data

Supplementary_Tables.docx Supplementary_Tables.docx
