# Supplementary material for: Neural substrates of brand equity: applying a quantitative meta-analytical method for neuroimage studies
Source: Heliyon. 2022 Jun 10;8(6):e09702. doi: 10.1016/j.heliyon.2022.e09702 (PMC9207674; doi:10.1016/j.heliyon.2022.e09702)
Supplement: Supplementary_Tables.docx [file mmc1.docx]

Supplementary Tables

**Supplementary Table S1**. **Studies included in the meta-analysis (Brand equity related brain regions).**

| **Experiment** | **Subject** | **Foci** | **Experiment stimuli** | **Detailed information** |
| --- | --- | --- | --- | --- |
| Erk et al. (2002) | 12 | 7 | Package images | Cultural objects (cars; sports car/ limousines / small cars) |
|  |  | 2 |  |  |
|  |  | 2 |  |  |
|  |  | 2 |  |  |
| McClure et al. (2004) | 16 | 7 | Brand logos (Coke / Pepsi) | Culturally Familiar Drinks |
| Deppe et al.(2005a) | 21 | 5 | Brand logos | Magazines |
| Deppe et al.(2005b) | 22 | 18 | Package images with brand logos | Beer / Coffee |
|  |  | 8 |  |  |
|  |  | 11 |  |  |
| Schaefer et al. (2006) | 13 | 1 | Brand logos | Culturally familiar brands（Cars) |
| Yoon et al. (2006) | 19 | 1 | Brand names/Adjectives | Various business sectors |
| Plassmann et al. (2007) | 22 | 1 | Brand logos (clothing images) | Department store |
| Schaefer & Rotte (2007a) | 13 | 5 | Brand logos | Cars |
| Schaefer & Rotte (2007b) | 14 | 5 | Brand logos | Cars |
|  |  | 1 |  |  |
|  |  | 5 |  |  |
|  |  | 2 |  |  |
| Plassmann et al. (2008) | 20 | 8 | Wine | Distinct prices wine (high prices and low prices) despite of same wines |
|  |  | 10 |  |  |
|  |  | 12 |  |  |
|  |  | 6 |  |  |
|  |  | 2 |  |  |
|  |  | 5 |  |  |
|  |  | 8 |  |  |
|  |  | 1 |  |  |
| Klucharev et al. (2008) | 18 | 10 | Package images without brand logos | Clothes, cosmetics, packaged food, etc. |
|  | 18 | 7 |  |  |
|  | 16 | 4 |  |  |
|  | 18 | 4 |  |  |
| Kato et al. (2009) | 40 | 18 | Advertising with brand logos | Coke Ad / Pepsi Ad |
|  |  | 8 |  |  |
|  |  | 3 |  |  |
|  |  | 26 |  |  |
| Reimann et al. (2011) | 16 | 1 | Musicians and titles |  |
|  |  | 2 |  |  |
| Casarotto et al. (2012) | 15 | 3 | Brand logos | 13 different product categories (clothing, transport, food, gas, cigarettes, etc.) |
|  |  | 1 |  |  |
|  |  | 19 |  |  |
| Esch et al. (2012) | 15 | 2 | Brand names and logos | 8 strong brands (BMW, Coca Cola, etc) and 8 weak brands (Kia, Yahoo, etc.) and 8 unfamiliar brands |
|  |  | 2 |  |  |
|  |  | 4 |  |  |
|  |  | 2 |  |  |
|  |  | 4 |  |  |
| Murawski et al. (2012) | 13 | 11 | Brand logos | Cups with Apple logo / Cups |
|  |  | 5 |  |  |
|  |  | 2 |  |  |
|  | 9 | 1 |  |  |
|  | 13 | 1 |  |  |
|  | 11 | 4 |  |  |
|  | 13 | 5 |  |  |
| Reimann et al. (2012) | 16 | 6 | Brand names and logo | Disney, Google, Apple and Starbucks Coffee. Etc. |
| Grabenhorst et al. (2013) | 13 | 2 | Fooods with Taste label / Health label |  |
|  |  | 4 |  |  |
|  |  | 2 |  |  |
|  |  | 2 |  |  |
|  |  | 1 |  |  |
|  |  | 3 |  |  |
|  |  | 4 |  |  |
| Bruce et al. (2014) | 17 | 7 | Brand logos | Foods (60), pizzahut, kfc, etc./ non-foods (60), lego, spongebob, windows, etc. |
|  |  | 5 |  |  |
|  |  | 4 |  |  |
| Burger and Stice (2014) | 9 | 10 | Product with logo | Coke Ad / Non-food Ad |
|  | 25 | 12 | Product & logo ad |  |
|  |  | 37 |  |  |
|  |  | 8 |  |  |
|  |  | 11 |  |  |
|  |  | 10 |  |  |
|  |  | 4 |  |  |
| Chen et al (2015) | 17 | 23 | Brand logo | 44 brands listed by the interbrand brand ranking |
| Enax et al. (2015) | 40 | 9 | Food images with FT certified marks | Various food category (chocolate, coffee, rice, etc..) |
|  |  | 5 |  |  |
|  |  | 7 |  |  |
|  |  | 10 |  |  |
|  |  | 4 |  |  |
|  |  | 4 |  |  |
|  |  | 6 |  |  |
|  |  | 3 |  |  |
|  |  | 2 |  |  |
| Audrin et al. (2017) | 38 | 20 | Items and brand logo images | Luxurious and non-luxurious (scarves, handbags, belts and purses) |
|  |  | 26 |  |  |
|  |  | 2 |  |  |
|  |  | 2 |  |  |
| Javor et al. (2018) | 37 | 0 | Brand logos | 15 familiar and 15 unfamiliar logos; BMW, Fiat, etc. |
| Jung et al. (2018) | 34 | 21 | Food images with logo (social / conventional enterprises) | Confectionery (i.e., cookies, chocolate, bread, and Korean traditional rice cake) |
|  |  | 15 |  |  |
|  |  | 9 |  |  |
|  |  | 8 |  |  |
|  |  | 11 |  |  |
|  |  | 4 |  |  |
|  |  | 4 |  |  |
|  |  | 25 |  |  |
|  |  | 1 |  |  |
|  |  | 21 |  |  |

**Abbreviations;** FT, fairtrade

**Reference;**

Audrin, C., Ceravolo, L., Chanal, J., Brosch, T., Sander, D., 2017. Associating a product with a luxury brand label modulates neural reward processing and favors choices in materialistic individuals. Scientific reports 7, 16176. https://doi.org/10.1038/s41598-017-16544-6

Bruce, A.S., Bruce, J.M., Black, W.R., Lepping, R.J., Henry, J.M., Cherry, J.B.C., Martin, L.E., Papa, V.B., Davis, A.M., Brooks, W.M., 2014. Branding and a child’s brain: an fMRI study of neural responses to logos. Social cognitive and affective neuroscience 9, 118–122. https://doi.org/10.1093/scan/nss109

Burger, K.S., Stice, E., 2014. Neural responsivity during soft drink intake, anticipation, and advertisement exposure in habitually consuming youth. Obesity 22, 441–450.

Casarotto, S., Ricciardi, E., Romani, S., Dalli, D., Pietrini, P., 2012. Covert brand recognition engages emotion-specific brain networks. Archives italiennes de biologie 150, 259–273. https://doi.org/10.4449/aib.v150i4.1478

Chen, Y.-P., Nelson, L.D., Hsu, M., 2015. From “where” to “what”: distributed representations of brand associations in the human brain. Journal of Marketing Research 52, 453–466. http://dx.doi.org/10.1509/jmr.14.0606

Deppe, M., Schwindt, W., Kraemer, J., Kugel, H., Plassmann, H., Kenning, P., Ringelstein, E.B., 2005a. Evidence for a neural correlate of a framing effect: Bias-specific activity in the ventromedial prefrontal cortex during credibility judgments. Brain research bulletin 67, 413–421. https://doi.org/10.1016/j.brainresbull.2005.06.017

Deppe, M., Schwindt, W., Kugel, H., Plassmann, H., Kenning, P., 2005b. Nonlinear responses within the medial prefrontal cortex reveal when specific implicit information influences economic decision making. Journal of Neuroimaging 15, 171–182. https://doi.org/10.1111/j.1552-6569.2005.tb00303.x

Enax, L., Krapp, V., Piehl, A., Weber, B., 2015. Effects of social sustainability signaling on neural valuation signals and taste-experience of food products. Frontiers in Behavioral Neuroscience 9, 247. https://doi.org/10.3389/fnbeh.2015.00247

Erk, S., Spitzer, M., Wunderlich, A.P., Galley, L., Walter, H., 2002. Cultural objects modulate reward circuitry. Neuroreport 13, 2499–2503. https://doi.org/10.1097/00001756-200212200-00024

Esch, F.R., Möll, T., Schmitt, B., Elger, C.E., Neuhaus, C., Weber, B., 2012. Brands on the brain: What happens neurophysiologically when consumers process and evaluate brands. Journal of Consumer Psychology 22, 75–85. https://doi.org/10.1016/j.jcps.2010.08.004

Grabenhorst, F., Schulte, F.P., Maderwald, S., Brand, M., 2013. Food labels promote healthy choices by a decision bias in the amygdala. Neuroimage 74, 152–163. https://doi.org/10.1016/j.neuroimage.2013.02.012

Javor, A., Kindermann, H., Koschutnig, K., Ischebeck, A., 2018. The neural correlates of trustworthiness evaluations of faces and brands: Implications for behavioral and consumer neuroscience. European Journal of Neuroscience 48, 2322–2332. https://doi.org/10.1111/ejn.14134

Jung, D., Sul, S., Lee, M., Kim, H., 2018. Social observation increases functional segregation between MPFC subregions predicting prosocial consumer decisions. Scientific reports 8, 1–13. https://doi.org/10.1038/s41598-018-21449-z

Kato, J., Ide, H., Kabashima, I., Kadota, H., Takano, K., Kansaku, K., 2009. Neural correlates of attitude change following positive and negative advertisements. Frontiers in Behavioral Neuroscience 3, 6. https://doi.org/10.3389/neuro.08.006.2009

Klucharev, V., Smidts, A., Fernández, G., 2008. Brain mechanisms of persuasion: how ‘expert power’modulates memory and attitudes. Social cognitive and affective neuroscience 3, 353–366. https://doi.org/10.1093/scan/nsn022

McClure, S.M., Li, J., Tomlin, D., Cypert, K.S., Montague, L.M., Montague, P.R., 2004. Neural correlates of behavioral preference for culturally familiar drinks. Neuron 44, 379–387. https://doi.org/10.1016/j.neuron.2004.09.019

Murawski, C., Harris, P.G., Bode, S., Egan, G.F., 2012. Led into temptation? Rewarding brand logos bias the neural encoding of incidental economic decisions. PloS one 7, e34155. https://doi.org/10.1371/journal.pone.0034155

Plassmann, H., Kenning, P., Ahlert, D., 2007. Why companies should make their customers happy: The neural correlates of customer loyalty. Advances in Consumer Researchces 34, 1–5.

Plassmann, H., O’doherty, J., Shiv, B., Rangel, A., 2008. Marketing actions can modulate neural representations of experienced pleasantness. Proceedings of the National Academy of Sciences 105, 1050–1054. http://dx.doi.org/10.1073/pnas.0706929105

Reimann, M., Castaño, R., Zaichkowsky, J., Bechara, A., 2012. How we relate to brands: Psychological and neurophysiological insights into consumer–brand relationships. Journal of Consumer Psychology 22, 128–142. https://doi.org/10.1016/j.jcps.2011.11.003

Reimann, M., Schilke, O., Weber, B., Neuhaus, C., Zaichkowsky, J., 2011. Functional magnetic resonance imaging in consumer research: A review and application. Psychology & Marketing 28, 608–637. https://doi.org/10.1002/mar.20403

Schaefer, M., Berens, H., Heinze, H.-J., Rotte, M., 2006. Neural correlates of culturally familiar brands of car manufacturers. Neuroimage 31, 861–865. https://doi.org/10.1016/j.neuroimage.2005.12.047

Schaefer, M., Rotte, M., 2007a. Favorite brands as cultural objects modulate reward circuit. Neuroreport 18, 141–145. https://doi.org/10.1097/wnr.0b013e328010ac84

Schaefer, M., Rotte, M., 2007b. Thinking on luxury or pragmatic brand products: Brain responses to different categories of culturally based brands. Brain research 1165, 98–104. https://doi.org/10.1016/j.brainres.2007.06.038

Yoon, C., Gutchess, A.H., Feinberg, F., Polk, T.A., 2006. A functional magnetic resonance imaging study of neural dissociations between brand and person judgments. Journal of Consumer Research 33, 31–40. http://dx.doi.org/10.1086/504132

**Supplementary Table S2. FSN robustness assessment for significant ALE maps of brand equity related brain regions.**

| **679 foci, 94 experiments, 2112 subjects, minimum FSN = 28** | | | | | | | | |
| --- | --- | --- | --- | --- | --- | --- | --- | --- |
| **Cluster #** | **Label (Side/Region/BA)** | **Peak voxel coordinates (MNI)** | | | **ALE**  **values** | **Cluster Size**  **(mm^3^)** | **Contributing**  **studies(k)** | **FSN** |
|  |  | **x** | **y** | **z** |  |  |  |  |
| 1 | L / Anterior Cingulate (VMPFC) / BA32 | -4 | 42 | -16 | 0.046 | 6368 | 27 | 270 |
| 2 | R / PHG (Entorhinal cortex) / BA28 | 18 | -4 | -16 | 0.036 | 2216 | 12 | 120 |
| 3 | L / Caudate Head (VS) | -6 | 12 | -4 | 0.034 | 1936 | 11 | 92 |
| 4 | R / Posterior Cingulate (RS region) / BA30 | 6 | -52 | 16 | 0.026 | 1064 | 9 | 15 |
| 5 | L / Lingual Gyrus / BA18 | -18 | -74 | -4 | 0.033 | 1032 | 7 | 13 |

FSN, Fail-Safe N analysis; ALE, activation likelihood estimation; BA, Brodmann area; MNI, Montreal Neurological Institute; R, right; L, left; VMPFC, ventral medial prefrontal cortex; PHG, parahippocampal gyrus; VS, ventral striatum; RS, retrosplenial.

**Supplementary Table S3. Differences between actual cluster and spherical ROI sizes**

| **Conjunction Analysis (Brand equity & DMN)** | | | |
| --- | --- | --- | --- |
| **Cluster #** | **Actual Cluster Size (mm^3^)** | **Spherical ROI** | |
|  |  | **Size (mm^3^)** | **Radius(mm)** |
| 1 | 1752 | 1751.640 | 7.478 |
| 2 | 1616 | 1615.488 | 7.279 |
| 3 | 320 | 319.968 | 4.243 |
| 4 | 288 | 287.851 | 4.096 |
| 5 | 24 | 23.983 | 1.789 |
| **Contrast Analysis (Brand equity > DMN)** | | | |
| **Cluster #** | **Actual Cluster Size (mm^3^)** | **Spherical ROI** | |
|  |  | **Size (mm^3^)** | **Radius(mm)** |
| 1 | 13608 | 13606.730 | 14.810 |
| 2 | 5760 | 5759.741 | 11.120 |
| 3 | 5456 | 5454.519 | 10.920 |
| 4 | 3384 | 3383.431 | 9.313 |
| 5 | 3144 | 3143.0420 | 9.087 |
| 6 | 2808 | 2807.124 | 8.751 |
| 7 | 2120 | 2119.825 | 7.969 |
| 8 | 2096 | 2095.974 | 7.939 |
| 9 | 2016 | 2015.449 | 7.836 |
| 10 | 1976 | 1975.591 | 7.784 |
| 11 | 1664 | 1663.902 | 7.351 |
| 12 | 224 | 223.911 | 3.767 |
| 13 | 104 | 103.967 | 2.917 |
| **Contrast Analysis (Brand equity < DMN)** | | | |
| **Cluster #** | **Actual Cluster Size (mm^3^)** | **Spherical ROI** | |
|  |  | **Size (mm^3^** | **Radius(mm)** |
| 1 | 9184 | 9183.671 | 12.991 |
| 2 | 2152 | 2151.906 | 8.009 |
| 3 | 912 | 911.581 | 6.015 |
| 4 | 640 | 639.993 | 5.346 |
| 5 | 560 | 559.907 | 5.113 |
| 6 | 224 | 223.911 | 3.767 |
| 7 | 200 | 199.862 | 3.627 |
| 8 | 184 | 183.939 | 3.528 |

To display in “Spherical ROI”, the number was truncated to three decimal places.
